# Perceptions of cultural and provisioning ecosystem services and human wellbeing indicators amongst indigenous communities neighbouring the greater limpopo transfrontier conservation area

**DOI:** 10.1016/j.heliyon.2024.e41448

**Published:** 2024-12-25

**Authors:** N.A. Nyathi, W. Musakwa, D.M. Azilagbetor, N.J. Kuhn

**Affiliations:** a Physical Geography and Environmental Change Research Group, Department of Geography and Physical Sciences, Faculty of Philosophy and Natural Sciences, University of Basel, Basel, 4056, Switzerland; b Department of Geography and Environmental Management and Energy Studies, University of Johannesburg, Auckland Park, Johannesburg, 2093, South Africa; c Institute for Biomedical Ethics, University of Basel, 4056, Basel, Switzerland

**Keywords:** Transboundary conservation, Ecosystem services, Ecosystem perceptions, Indigenous people

## Abstract

Nature plays a crucial role in providing ecosystem services (ESs) essential for human wellbeing and biodiversity conservation in rural areas. However, existing paradigms often lack an integrative approach towards rural livelihoods and wellbeing, highlighting the need for a comprehensive understanding of the relationship between human wellbeing (HWB) and ESs. The area around the Greater Limpopo Transfrontier Conservation Area (GLTFCA) offers such ESs to indigenous people who rely heavily on these natural resources. Thus, this study aimed to quantify indigenous people's perceptions of cultural and provisioning ESs and their link to human wellbeing in villages adjacent to the GLTFCA. Key informant interviews were initially conducted to identify ESs as perceived by the participants. Subsequently, face-to-face surveys were carried out in 9 wards across South Africa and Zimbabwe, involving 350 participants. Respondents were asked about the availability of ESs, their levels of degradation, drivers of change, and the impact on their wellbeing. Responses were captured using Likert scales, and multiple regression models analysed the relationships between socio-demographic characteristics and ESs. Results indicated that indigenous people perceived both cultural and provisioning ESs to be available but degrading, with provisioning services degrading more rapidly. Climate change, legislation/policies, and poverty were identified as key drivers of this change. Socio-demographic factors such as gender, nativeness, and employment level influenced perceptions of both ESs. Overall, participants reported that both ESs contribute to their human wellbeing and livelihoods through life satisfaction, happiness, living standards, safety, security, and good health. Finally, this study's findings uniquely offer a baseline for these ESs accounting, demonstrating their direct and indirect benefits to indigenous communities' livelihoods and well-being.

## 1 Introduction

Ecosystem services (ESs) are the contributions of natural systems to human wellbeing [1]. These services provide material and non-material benefits to people for their livelihoods and wellbeing [1]. In 2005, the Millennium Ecosystem Services Assessment (MEA) broke down ESs into four categories: *provisioning*— which includes food, fresh water, fibre, and fuel [2]; *regulating*— such as climate and disease control [3]; supporting— including nutrient cycling and primary production [4], and *cultural*—providing religious factors, education and aesthetic [5]. Of these categories, MEA (2005) [6] reported that provisioning and cultural services are the least accessible ESs to rural communities worldwide, even though local people survive on them. Furthermore, their availability is linked to environmental stressors such as land degradation, invasive species [7] and increases in human population [8], thus affecting livelihoods and wellbeing.

Natural systems such as protected areas are pivotal in supplying ESs crucial for human wellbeing and biodiversity conservation in rural areas [9]. Their conception, functions and processes within ecological paradigms are widely accepted in biodiversity management [10]. However, they often lack an integrative approach with rural livelihoods that are located adjacent to southern African protected areas and wellbeing for nature-based solutions. Currently, about 12 % of arable land in Southern Africa is designated to protected areas, most of which lie in rural southern Africa [11]. While these protected areas provide ESs, their establishment in transboundary areas inevitably affect the benefits indigenous people get from ESs resulting in significant conflict and altered perspectives of them [12]. Human wellbeing (HWB) is defined by [2] as a human experience that includes the basic materials for a good life, freedom, health, a sense of belonging, social cohesion, happiness, sense of security, and cultural and spiritual fulfilment. Furthermore, it has long been established that HWB is intricately linked to nature through the provision of ecosystem services (ESs), which is a key reason for the integration of ESs into policy [13,14]. Additionally, policies such as the MEA, the United Nations' Sustainable Development Goals (SDGs) and the Intergovernmental Science-Policy on Biodiversity and Ecosystem Services (IPBES) recognise the ESs concept as fundamental to indigenous people's livelihoods and wellbeing when pursuing sustainable development [4]. Consequently, the IPBES, SDGs and MEA have identified goals and targets towards taking a people-and-nature-approach for developing indigenous rural communities. However, progress towards these goals is slow and needs urgent advancements [4].

Reviews by Refs. [14,15] found that in southern Africa most rural communities, especially surrounding transboundary conservation areas, rely heavily on both provisioning and cultural services that are mostly degraded. This has a negative impact on indigenous peoples' wellbeing, posing a challenge towards achieving the SDGs. Likewise [16], suggested that contextual knowledge about the importance of provisioning and cultural ESs and their impacts on HWB would benefit rural communities living in the vicinity of protected areas of Ghana. However, as noted by [[17], [18]], most studies still do not prioritize local communities' perceptions on ESs in policy development, thus resulting in a narrow range in policies to address ecosystem-based rural livelihoods and wellbeing [19]. Additionally [20], noted that ESs research in developing countries show little to no evidence of empirical data that incorporates local communities' perceptions for socio-ecological development. For example, in the Western Himalayan Gurez valley in Pakistan [21], analysed attitudes towards provisioning ecosystem services, climate change impacts, and local community perceptions of these. They found that additional data on climate and perceptions could better inform policy implementation for managing provisioning ESs in the area. Although their findings highlight the need to study ESs to progress their sustainable use, they do not include people's perceptions on cultural ESs necessary for socio-ecological development and HWB.

Diverse sources of knowledge in ESs assessments enhances practical conservation efforts [1] and provide evidence-based data to understanding ESs dynamics [7]. Furthermore, the general knowledge base on specific services and functions associated with well-functioning ecosystems is established and assessed by the IPBES and the International Union for Conservation of Nature (IUCN) [22]. Additionally, adopting a local community-centred approach to understanding perceptions of ESs promotes baseline policy formulation and help contribute to a good quality of life for rural communities [23], an approach still lacking in Southern Africa, specifically in the Greater Limpopo Transfrontier Conservation Area (GLTFCA) [24]. While this approach adequately addresses the nexus between ESs and local communities in Southern African protected areas [25], it is also acknowledged that factors such as national politics [26], localised land degradation [27], microclimates [28] and human-wildlife conflict [29] shape individual perceptions of ESs [30]. While many studies focus on ecological and environmental factors in ecosystem services (ESs) conservation, few integrate local people's perspectives, which are essential for effective, community-driven conservation strategies. Without these perspectives, recommendations risk promoting top-down policies that may not align with local needs for human well-being [29].

To promote holistic ESs management strategies in the GLTFCA, it is essential to integrate indigenous people's perceptions of ESs into policy frameworks [31]. Our study uniquely offers a baseline for these ESs accounting, demonstrating their direct and indirect benefits to indigenous communities' livelihoods and well-being. Addressing a critical gap in research, as few studies have focused on transboundary Southern African protected areas, this study sought to present valuable insights into the current state of these ESs and their link to human well-being from the perspective of indigenous communities, a previously unexplored approach in this region. In this study, we hypothesised that indigenous people have favourable perceptions of ESs because they live within a transboundary protected area, and that these ESs contribute positively towards their wellbeing. This hypothesis is grounded in the MEA [6] and Nature's Contributions to People (NCP) frameworks [32], linking ESs [33] to human well-being (HWB) [34]. It integrates environmental perception theory and the Social-Ecological Systems (SES) [35] framework to explore how indigenous perceptions of provisioning and cultural ESs impact livelihoods and well-being in transboundary conservation areas. Thus, we aimed to both quantify and qualify people's perceptions towards the current state of cultural and provisioning ESs and their impact on indigenous people's wellbeing. To achieve this, our study aimed to.

(i) assess factors influencing local people's perceptions of the availability of cultural and provisioning ESs;
(ii) quantify the drivers of change for these ESs according to people's perceptions, and
(iii) link cultural and provisioning ESs with human wellbeing indicators according to indigenous people's perceptions.

## 2 Methods and Procedures

### 2.1 Study area

**Fig. 1:**
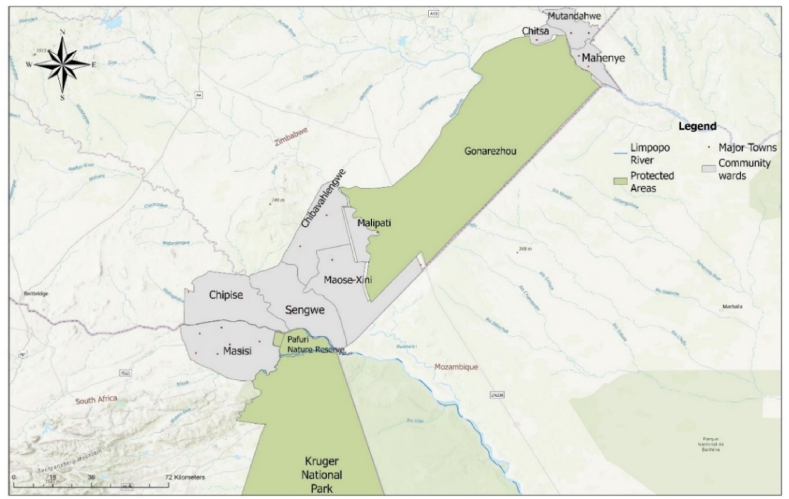
Location of the selected study sites around the GLTFCA.

The study site comprises of nine (9) community wards, six of which are adjacent and located north of the Kruger National Park, South Africa and south of the Gonarezhou National Park, Zimbabwe (Fig. 1) and three wards are located adjacent to and north of the Gonarezhou National Park. The area is in a lowland Savannah ecosystem with an altitude ranging from 107 m above sea level to its peak at 838 m [24]. This area has a seasonal semi-arid climate with rainfall between November and March, and May to September are its driest months [36]. Average annual rainfall ranges from 243 mm to 648 mm and the average temperature extends from a minimum of 25 °C to a maximum of 38 °C.

### 2.2 Site selection

The study was undertaken within the Vhembe biosphere reserve in South Africa (Masisi, Bende Mutale, Dovho villages), and the northern Chiredzi district (Chitsa, Mahenye & Mutandahwe) and southern Chiredzi district (Chipise, Chibavahlengwe, Malipati, Maose-Xini & Sengwe) in Zimbabwe (Fig. 1). These communities were purposefully selected because they are located adjacent to the two major protected areas that form part of the Greater Limpopo Transfrontier Conservation Area (GLTFCA) and on the land where local indigenous people were forcibly removed for conservation reasons [37]. Additionally, these sites form part of the GLTFCA conglomerate, which was established as part of a peace treaty between the two countries, to create environmental corridors in which animals could roam freely [38] and promote vegetation regeneration [39]. This framework, developed by the Peace Parks Foundation [40], further aimed to support socio-ecological conservation and the establishment of rural livelihoods. Additionally, this area houses more than 16 % of total agricultural land in Southern Africa, responsible for provisioning ESs [41]. In total, 43 sub-villages (Appendix 1, Table A1) were represented in the sample collected using a stratified random sampling technique as recommended for villages that are located far from cities, under-developed and not easy to access [42].

### 2.3 Survey data collection

**Fig. 2:**
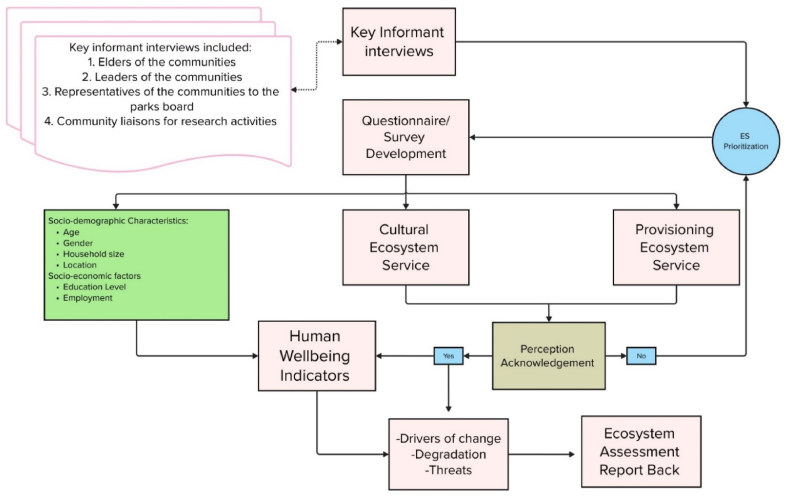
Schematic chart of the methodology used in this study.

Data were collected through a questionnaire survey (Appendix 2) that was developed following key informant responses, as recommended by [24,43]. Prior to developing the questionnaire, a pilot study was conducted in February 2020, where ten (10) key informants, comprising of community leaders, park officials, respected elders and other community representatives were consulted (Fig. 2). To identify key informants, we first established community entry by consulting the chiefs of both communities in South Africa and Zimbabwe. In each location, we coordinated with local parks management, who introduced us to community leaders and liaisons as well as rangers from the parks. From there, we used a snowball sampling technique, where these initial contacts guided us to other important community leaders and key informants. This approach ensured that we engaged with individuals who held valuable insights and influence within their communities.

These key informants were chosen because of their vast knowledge of the study sites and their established relationships with community members [44,45]. Furthermore, they were distinctly chosen for their active participation in community visioning activities for socio-ecological framework and policy development as well as the enhancement of ESs knowledge. Additionally, these key informants were chosen because they represented community members as they incorporated both provisioning and cultural ESs knowledge into their daily activities. Key informant interviews required that respondents rank, in order of importance, ESs obtained from the MEA 2005 report and the IPBES global report on biodiversity and ecosystem services for rural livelihoods [22]. Additionally, they were asked to explain the various ways in which they perceive ecosystem changes, degradation and threats, and the impact these ESs have on their well-being. Additional questions inquired about impacts of the ESs of aesthetics, culture, religion and access to provisions on the communities’ livelihoods and mental well-being.

The questionnaire was administered once in 2021 and once in 2022 because the sites were inaccessible due to COVID-19 travelling restrictions. On both occasions, a 3-day workshop was held where field assistants appointed to collect data were trained on research ethics, consent and methods [46]. The training included translating the questions into the local languages such as Xitsonga, Tshivenda, IsiNdebele and Shona, to enable a deeper understanding of the questions and responses for respondents. A workshop was also held with the assistants after the fieldwork was completed, to confirm responses and for quality management, including checking errors. The field assistants comprised mainly of females as part of community development and gender mainstreaming, as recommended by the United Nations Women Charter [47]. Ethical clearance was obtained from the University of Johannesburg (Ethics reference number: 2021-04-01/Nyathi_Musakwa) and each respondent had to sign a consent form prior to participation. Each questionnaire took approximately 25 min, and data collected was kept strictly confidential and anonymous.

The questionnaire collected the following data: socio-demographics, cultural ESs, provisioning ESs, basic needs, and human wellbeing (Appendix 1, Table A2). Each respondent was asked to acknowledge awareness of ESs, their changes and drivers of change, degradation rates and threats, and how these impacted his/her livelihoods [42]. Respondents were requested to rank each ESs according to degradation rate, on a four-point Likert scale with options from “*not degraded*”, “*somewhat degraded*”, “*degraded*”, and “*severely degraded*”. For threats, respondents were asked to point out which ESs were threatened in the future. Furthermore, respondents were asked to give an account of their perceptions of well-being and point out which ESs had a “negative”, “no”, “slight”, “high” and “extreme” impact towards their wellbeing (Table 1). Scales of the other variables included in the questionnaire can be found in Appendix 1, Tables A3a and A3b. Respondents who did not understand the concept of ESs were asked to list and then prioritize the order in which they had access to ecosystem goods and services. Thereafter, the individual collecting the data determined which category of ESs it belonged to (Fig. 2). The limitation with this approach is that where an ESs was not mentioned, it was not clear whether it was because the respondent lacked awareness of the ESs, or if their responses were indeed negative, or simply because the service did not exist. This is consistent with the challenges that [19] faced where they tried to raise awareness in local communities about the value of ESs to human wellbeing.

**Table 1:** Four and five-point Likert scale decision interval for ESs perceptions and human wellbeing domains.

Likert scale	Interval for mean value	Decision for degradation	Decision for human-wellbeing
1	1.00–1.75	Not degraded (not important)	Negative impact (negative influence)
2	1.76–2.50	Somewhat degraded (slightly important)	No Impact (no influence)
3	2.51–3.25	Degraded (Highly important)	Potential Impact (slight influence)
4	3.26–4.00	Severely degraded (Extremely important)	Minor Impact (high influence)
5	4.01–5.00	∗	Major Impact (Extreme influence)

### 2.4 Data analysis

Data collected from the key informants were analysed using prioritisation and narrative methods. Further, the MAXQDA software was used to analyse qualitative data from key informant interviews, prioritizing cultural and provisioning ESs for the development of the questionnaire. Quantitative data were captured using Likert scales and analysed using R, while qualitative insights were processed in MAXQDA to ensure a comprehensive understanding. Data were initially clustered by country to facilitate comparison across regions. Within each country, data were clustered by the ESs identified and the respondents' gender and ages [19,49]. Additionally, data obtained from the four-point Likert scale were analysed using descriptive statistics including percentage, frequency, mean and standard deviation according to the ESs variables of degradation perceptions and human wellbeing indicators. Multiple regression analysis was performed to identify the determinant factors of cultural and provisioning ESs, degradation and changes and threats in the regression analysis [3]. Furthermore, 6 socioeconomic and ESs-related variables were included, and their influence on indigenous people's perceptions regarding their wellbeing was assessed [3]. Finally, multiple regression was used to test the relationship between ESs, demographics and HWB indicators.

### 2.5 HWB domains

HWB remains poorly understood in the context of the ESs in rural areas due to the complexities surrounding policy frameworks, equity, inclusivity, feasibility and lack of adequate terminology [50]. To reliably measure indigenous people's perspectives of the role that ESs have on their wellbeing, HWB domains were established through measurable objective and subjective parameters established from questionnaire surveys by Ref. [51]. These domains were established because of their robust enquiry and holistic view of the impacts cultural and provisioning ESs have on individual HWB in an economic and environmental perspective for basic human needs.

Here, a total of 50 publications were systematically reviewed to retrieve HWB indices for rural development. Additionally, MAXQDA was incorporated to give weights to the different HWB indicators according to domains that were established from open-ended responses provided by respondents (Appendix 1, Table A8). The weight assigned to the indicators was then determined for importance within people's perceptions for their livelihoods and wellbeing. From this, a generic diagram of HWB domains and their link to ESs impact was derived based on spatial scales and domain frequency.

## 3 Results

### 3.1 Demographics

Out of 360 survey participants, only 350 responses were analysed, with the sample consisting of 44 % males, 51 % females, and 6 % preferring not to disclose their gender (Table 2). The majority of respondents from Zimbabwe were female with an average age of 36 while the minority consisted of males from South Africa that are aged 65 and above. There were more females that were aged between 19 and 37 in south Africa (52 %) than in Zimbabwe (47 %). Notably, 74 % of respondents were unemployed, with the majority of the unemployed being the primary earners aged 26–54, and 47 % had only secondary education (Table 2).

**Table 2:** Sample composition of Sociodemographic factors of the survey respondents.

		South Africa	Zimbabwe	Total	Percentage
**Gender**	Men	72	85	157	**45**
	Women	90	89	179	**51**
	Prefer Not to Say	13	1	14	**4**
**Age**	19–37	75	68	143	**41**
	38–54	64	82	146	**42**
	55–65	25	15	40	**11**
	65>	11	10	21	**6**
**Origin**	Native	129	156	285	**81**
	Non-Native	46	19	65	**19**
**Household Size**	1–3	30	14	44	**13**
	4–5	84	47	131	**37**
	5>	61	114	175	**50**
**Education**	Primary	29	61	90	**26**
	Secondary	96	69	165	**47**
	College/TVET	15	16	31	**9**
	University	13	6	19	**5**
	None	22	23	45	**13**
**Employment**	Employed	22	12	34	**10**
	Self-Employed	33	23	56	**16**
	Unemployed	120	140	260	**74**

### 3.2 Perceptions of cultural and provisioning ESs degradation and threats

Cultural ESs were listed as cultural heritage, ecological knowledge, traditional knowledge, scientific research, education/interpretation, landscape aesthetic, tourism/ecotourism and recreation. Additionally, provisioning services were listed as fodder and forage, wild plants, freshwater, soils, traditional medicine, timber, livestock and crops (Appendix 1, Tables A3a and A3b). Here, 82 % (n = 287) of the respondents believed that cultural ESs have declined over the years and 80 % (n = 281) of the respondents thought that the cultural ESs are threatened. On the other hand, 87 % (n = 303) of the respondents believed that provisioning ESs have declined and 85 % (n = 296) perceived that these are threatened (Appendix 1, Fig. A1).

Overall, results show that both cultural and provisioning services were perceived as available, degraded and threatened (Appendix 1, Fig. A3). Within cultural ESs, community members perceived recreation, ecological knowledge and traditional knowledge as highly important for them and severely degraded (m ≥ 2.85) (Table 3). Additionally, respondents perceived education and scientific research to be important but degraded. Although this was the general consensus about education, scientific research showed a high standard deviation (1.01), indicating a range of opinions among respondents thus suggesting different views of its degradation status. Within provisioning services, results show that wild plants, timber and fodder-and-forage are perceived to be severely degraded (m ≥ 2.85). Community members also acknowledged that crops, freshwater, traditional medicine and livestock are highly important for them, but are degraded. Standard deviation (1.14) for crops showed high variability, indicating a wide range of opinions among respondents, suggesting differing views on their degradation status (Table 3).

**Table 3:** Perceptions of ES degradation for both cultural and provisioning services.

		ND (%)	SD (%)	D (%)	SeD (%)	Mean	Std. Dev∗	Var∗	N∗
Cultural	Cultural Heritage	20	27	41	12	2.44	0.94	0.89	328
	Ecological Knowledge	11	18	45	26	2.86	0.93	0.87	328
	Education/Interpretation	23	27	37	14	2.42	0.99	0.97	327
	Landscape/Aesthetic	10	37	37	16	2.59	0.87	0.76	326
	Recreation	13	20	36	31	2.86	1	1	326
	Scientific Research	22	24	37	16	2.47	1.01	1.03	326
	Tourism/Ecotourism	13	26	43	18	2.66	0.92	0.84	327
	Traditional Knowledge	8	24	41	26	2.85	0.9	0.81	329
Provisioning	Crops	23	16	31	31	2.69	1.14	1.29	332
	Fodder&Forage	15	25	46	15	2.79	0.98	0.95	332
	Freshwater	12	22	41	25	2.67	0.96	0.93	330
	Livestock	12	24	37	27	2.64	0.86	0.74	332
	Soils	15	25	40	21	2.60	0.91	0.82	332
	Timber	13	21	40	26	2.79	0.95	0.90	332
	Traditional Medicine	13	25	38	24	2.73	0.97	0.93	331
	Wild Plants	12	25	50	13	2.80	0.97	0.94	331

People's perceptions were captured using a four-point Likert scale and analysed on R, not degraded (ND), somewhat degraded (SD), degraded (D) and severely degraded (SeD). Overall perception was decided following the recommendation summary range as prescribed by [48]. Std Dev∗ is Standard deviation, Var∗ is variance and N∗ is the total number of valid responses in the survey.

For both services, individual multiple regression analyses results can be found in Appendix 1, Tables A6 and A7. Factors such as country (Zimbabwe; *p* = <0.001), gender (female; *p* = < 0.001), employment level (*p* = <0.001) and household size (*p* = 0.007) influence the perceptions of provisioning services availability and degradation. The p-values for these variables indicate that the coefficient is statistically significant from 0, which means that there is evidence from the sample that females from Zimbabwe and overall household size impact people's perceptions of provisioning services. Factors such as being in South Africa (p = 0.009), being male (p = 0.04), female (p = 0.013), or preferring not to disclose gender (p = 0.05), and employment level (p = 0.007) significantly affect perceptions of cultural services availability and degradation (Table 4).

**Table 4:** Multiple regression coefficients between Human Wellbeing variables and sociodemographic factors where *t* is the test statistic and *p* represent the significance value.

**Provisioning Services**								**Cultural Services**						
	**B**	**Unstanda-rdized Coefficient**s	**Standar-dized coefficients**	**t**	**p**	**95 % confidence interval for B**		**B**	**Unstanda-rdized Coefficients**	S	**t**	**p**	**95 % confidence interval for B**	
		**Beta**	**Standard** **Error**			**Lower** **Bound**	**Upper Bound**		**Beta**	**Standard** **Error**			**Lower Bound**	**Upper Bound**
**(Constant)**	1.25		0.67	1.86	0.064	−0.08	2.58	2.98		0.66	4.52	<0.001	1.67	4.28
**Country 1 (South Africa)**	−0.32	−0.09	0.21	−1.55	0.122	−0.73	0.09	−0.16	−0.05	0.18	−0.89	0.009∗	−0.51	0.19
**Country 2 (Zimbabwe)**	−0.8	−0.24	0.19	−4.17	<:0.001 ∗	−1.18	−0.42	0.06	0.02	0.18	0.33	0.73	−0.3	0.42
**Gender 1 (Male)**	0.65	0.19	0.51	1.29	0.198	−0.35	1.66	0.34	0.12	0.17	2.02	0.04∗	0.01	0.67
**Gender 2 (Female)**	0.78	−0.22	0.21	−3.81	<:0.001 ∗	−1.19	−0.38	0.26	0.09	0.44	0.6	0.01 ∗	−0.61	1.14
**Gender 3 (Prefer not to say)**	−0.47	−0.05	0.51	−0.93	0.354	−1.47	0.53	0.83	0.11	0.42	1.96	0.05∗	−0.01	1.66
**Age**	0.01	0.02	0.04	0.27	0.791	−0.07	0.1	−0.02	−0.03	0.04	−0.47	0.635	−0.09	0.05
**Household Size**	0.23	0.16	0.09	2.7	0.007∗	0.06	0.4	−0.06	−0.05	0.07	−0.86	0.158	−0.21	0.08
**Education Level**	0.1	0.07	0.08	1.35	0.179	−0.05	0.25	0	0	0.06	0.04	0.969	−0.13	0.13
**Employment**	0.54	0.56	0.04	1.24	<:0.001 ∗	0.45	0.63	0.17	0.17	0.06	2.69	0.007∗	0.05	0.3

Provisioning ESs such as crop production and livestock directly benefit people's livelihood [52], with this, respondents named their crop production products as well as the livestock they rear for both personal and economic reasons. Survey results show that local people mainly grow and harvest maize (*Umbila/Nsima*), cabbage, okra, onions, string beans, sugar cane, sorghum, watermelons, kale, bananas, tomatoes, avocados, litchis and bell peppers. Additionally, they forage for wild indigenous fruits such as marula, smellyberry fingerleaf fruit (*Tsubvu*), monkey orange, bird plum (*Nyii*), macademia nuts, snot apples (*uXakuxaku/morojwa*), wild medlar (*Umviyo/Munjiro*), baobab fruit, red milkwood (*Nhlantswa/Mubululu*) and wild plum (*Mothekele/Umgwenya*). Livestock include cattle, goats, sheep, pigs, chickens and quails (Appendix 1, Table A5). Further results show that domestic crops are mostly used for consumption and selling while wild indigenous fruits are used mostly for selling. Community members further elaborated on additional uses for the different crops and fruits that include medicinal, religious purposes and aesthetics. As such, these ESs belong to both cultural and provisioning services.

### 3.3 Perceptions of ESs drivers

**Fig. 3:**
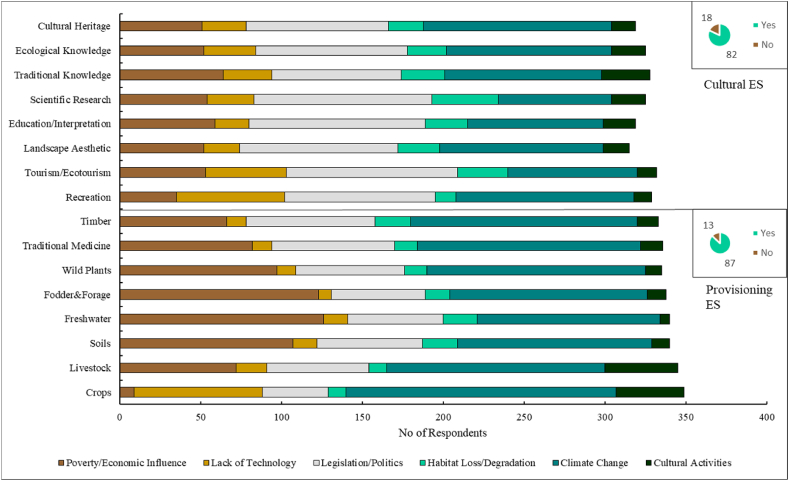
Perceptions factors driving ESs changes for cultural and provisioning ESs.

Perceptions of ESs drivers and their causes were quantified (Fig. 3). Results show that 82 % of the respondents agreed to cultural and provisioning (87 %) services declining. From the key informant interviews, drivers of ESs changes were identified as poverty/economic influence, lack of technology, legislation/policy, habitat loss/degradation, climate change and cultural activities (Appendix 1, Table A4). Respondents quantified the drivers of change against singular ESs of both cultural and provisioning. Amongst cultural ESs, results show that cultural heritage, ecological knowledge, recreation, and landscape aesthetic were perceived to be the most affected, with changes in each of them being attributed to climate change, legislation/politics and poverty. Individually, it is worth noting that scientific research and tourism were affected by the lack of technology while education and traditional knowledge were mostly affected by poverty. Additionally, scientific research, tourism/ecotourism and landscape aesthetic were perceived to be impacted by habitat loss/degradation. Since other cultural services are used for both religious and cultural activities, results here show that these activities affect traditional knowledge, ecological knowledge and tourism.

Regarding provisioning ESs, all the singular services are negatively affected. Among these, crops, livestock, soils, fodder & forage and timber are mainly affected by climate change, poverty and legislation/politics. Other singular services such as soils and freshwater are also mostly affected by legislation/politics. Additionally, lack of technology affects crop production, livestock and freshwater. Cultural activities were also quantified as drivers of crop production, livestock and fodder & forage. Within provisioning services, the main drivers of change are poverty/economic influence, climate change and legislation/politics (Fig. 3).

### *3.4* *Ecosystem services and human wellbeing indicator*s

Respondents were asked to give their perceptions of HWB indicators per singular ESs in an effort to link the two to establish HWB domains for rural livelihoods. In comparison, provisioning services had more responses than cultural services. High mean scores (>4.0) and low standard deviation here indicate a strong belief in and agreement within respondents that crops, livestock, soils and traditional medicine as have a major contribution to their wellbeing (Table 5). Although additional provisioning services such as fodder and forage, freshwater, timber and wild fruits do not have high mean values (≥3–4), results indicate a significant belief in the contribution of the service to individual wellbeing (Table 5). This means that respondents perceived all provisioning services to have a high to extremely high influence on their individual wellbeing and none had a negative influence (Appendix 1, Fig. A4).

**Table 5:** Perceptions of cultural and Provisioning ESs impact levels on human wellbeing.

		NeC (%)	NoC (%)	PC (%)	MiC (%)	MaC (%)	Mean	Std. Dev	Var	N
Cultural	Recreation	4	35	24	13	15	3.84	2.36	5.58	196
	Tourism/Education	11	27	32	16	13	3.86	1	1	326
	Landscape/Aesthetic	3	20	38	24	14	3.66	0.92	0.84	327
	Education/Interpretation	3	9	33	22	33	3.42	0.99	0.97	327
	Scientific Research	4	12	41	18	24	3.59	0.87	0.76	326
	Traditional Knowledge	1	9	18	28	36	4.47	1.01	1.03	326
	Ecological Knowledge	0	8	27	32	27	3.85	0.9	0.81	329
	Cultural Heritage	0	7	19	23	44	3.44	0.94	0.89	328
Provisioning	Crops	2	2	7	11	78	4.61	0.84	0.71	332
	Fodder&Forage	3	13	32	25	26	3.58	1.11	1.23	332
	Freshwater	3	3	20	17	56	4.21	1.06	1.13	332
	Livestock	1	2	8	10	79	4.66	0.77	0.59	331
	Soils	1	3	16	23	58	4.34	0.89	0.8	330
	Timber	2	8	19	22	50	4.1	1.07	1.15	332
	Traditional Medicine	3	10	20	20	47	3.98	1.16	1.34	332
	Wild Plants	5	12	30	29	24	3.55	1.14	1.3	331

People's perceptions on HWB were captured using a five-point Likert scale and analysed using R: negative contribution (NeC), no contribution (NoC), potential contribution (PC) and minor contribution (MC) and major contribution (MaC). Overall perception was decided following the recommendation summary range as prescribed by [48]. Std Dev∗ is Standard deviation, Var∗ is variance and N∗ is the total number of valid responses in the survey.

On cultural services, most community members reported that traditional knowledge (m = 4.47) has a major contribution towards their wellbeing. Additional cultural services such as tourism/education, landscape aesthetic, ecological knowledge and cultural heritage have a moderate contribution towards their wellbeing (Table 5). Scientific research and education had a wide range of opinions among respondents, indicating a moderately low significance. While recreation had a level of influence towards the wellbeing of the respondents, the high standard deviation (Std. Dev = 2.36) indicated highly varied opinions on its contribution (Table 5).

Results showing the relationship between sociodemographic factors and HWB variables indicate that access to food (*p* = <0.001), medical services (*p* = <0.001), basic education (*p* = 0.006), religious/spiritual values (*p* = <0.001), job creation (*p* = 0.016), life satisfaction (*p* = 0.04) and happiness (*p* = 0.07) have statistical significance and confidence that these factors affected the perceptions of the contribution of both categories of ESs to human wellbeing (Table 6).

**Table 6:** Multiple regression coefficients between Human Wellbeing variables and sociodemographic factors where *t* is the test statistic and *p* represent the significance value.

Socio-demographic Factors						
	Coefficient B	Standard error	z	p	Odds Ratio	95 % conf. interval
**Constant**	−2.48	0.51	4.89	<0.001∗	0.08	0.03–0.23
**Fresh water**	0.5	0.39	1.3	0.195	1.66	0.77–3.55
**Access to food**	−2.62	0.55	4.77	<0.001∗	0.07	0.02–0.21
**Medical services**	2.81	0.51	5.46	<0.001∗	16.55	6.05–45.3
**Basic education**	−1.03	0.37	2.75	0.006∗	0.36	0.17–0.74
**Religion/Spiritua Values**	1.57	0.36	4.35	<0.001∗	4.79	2.37–9.72
**Mental Well- Being**	0.34	0.31	1.1	0.269	1.41	0.77–2.57
**Job Creation**	0.66	0.27	2.4	0.016∗	1.93	1.13–3.29
**Life satisfaction**	0.34	0.17	1.98	0.047∗	1.4	1–1.96
**Hapiness Index**	0.3	0.17	1.79	0.073∗	1.35	0.97–1.88

**Fig. 4:**
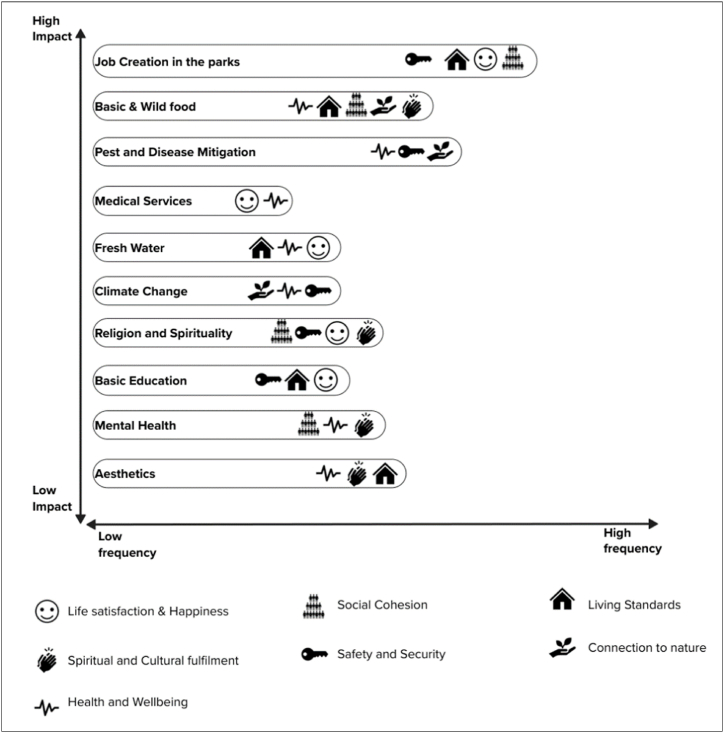
Human wellbeing domains for sustainable livelihoods for indigenous people in the GLTFCA.

HWB domains included impacts such as life satisfaction/happiness, social cohesion, living standards, spiritual and cultural fulfilment, safety and security, connection to nature and health and wellbeing (Appendix 1, Table A8). Results highlight that perceptions were weighted according to the impact they had per individual, and that none of these domains affected everyone equally. The HWB indicators that had the highest impact were job creation within the parks, basic & wild food, pest and disease mitigation, medical services and access to fresh water (Fig. 4). Mental health and aesthetics contributed the least towards human wellbeing. It is worth noting that this model does not emphasize the frequency of responses, but rather, the weight and impact each HWB indicator has on individuals [50]. For example, mental health, basic education and religion/spirituality had a high frequency of responses, but the lowest impact on livelihoods and human wellbeing. Medical services and pest and disease mitigation display a moderate frequency of responses but have a high impact on people's wellbeing (Fig. 4). This analysis is reflective of what people perceive as important for their livelihoods, as well as the ESs factors influencing their wellbeing directly on a daily basis.

## 4 Discussion

### 4.1 Ecosystem services availability, drivers and degradation

This study investigated the factors that shape how local people living within the vicinity of the Greater Limpopo Transboundary Conservation Area perceive cultural and provisioning ESs, their degradation, drivers of change and threats associated with future changes. Findings show that people are fully aware of the ESs that were presented to them but were not familiar with the scientific concept of the term. This is consistent with [9,37,51], who reported that people are normally aware of the ESs that directly benefit them, but not the scientific term. Our study also showed that there were differences in the levels of local people's perceptions of various ESs' categories, however, people are most aware of the cultural and provisioning services as they provide direct material and non-material benefits to meet their broader needs and desires. Among the many benefits they get from cultural and provisioning services are distinct monetary and spiritual values. These findings are different from Ref. [12] who found that residents at the Guanyin Mountain Nature Reserve in China perceived supporting, regulating, cultural and provisioning ESs. Additionally, indigenous people in our study perceived ESs to be present and available, but also degrading and under threat from various sources. This emphasizes the need for empirical research on perceptions of ESs for better ESs management.

Amongst cultural ESs, people perceived the most degraded individual services as recreation, traditional knowledge and ecological knowledge. This could be attributed to a sub-optimal state of local knowledge systems (LKS) that have traditionally sustained indigenous communities by accessing knowledge and passing it down to the next generations, as observed by [53]. These knowledge systems are tied to evolving ecological knowledge that allows local people to adapt to everchanging stresses in their environments [53]. Since rural people live in remote areas, knowledge about their environment helps them harness the potential of cultural ESs such as indigenous trees that supply them with both provisions and medicinal benefits [30]. For example, most people reported that knowledge about indigenous woody vegetation such as the *strychnos spinosa* (monkey orange), *berchemia discolour* (Nyii), *thespesia garckeana* (uXakuxaku), *mimusops zeyheri* (nhlantswa) and *prunus americana* (mothekele) help them treat ailments such as stomach/abdominal disease and skin conditions; treat snake bites; and improve the immune system (Appendix 1, Table A5), observations consistent with [54]. Other trees such as the *adansonia digitate* (baobab) and *vitex pooara* (tsubvu) are beneficial for recreation and spiritual purposes [55].

Perceptions of drivers of change for cultural services varied according to socio-demographic factors such as age, gender, education level, household size and nativity location. Overall, drivers of change were identified as legislation/politics, poverty/economic influence, climate change and lack of technology (Appendix 1, Table A4). This is consistent with the socio-demographic factors identified by [42] who found that respondents’ unemployment rate, household size and education level are some of the factors that affect perceptions of ESs change in low-income countries. Additionally, factors such as corruption, science and technology and habitat loss have been shown to influence the degradation of cultural services, leading to compromised livelihoods and the concept of *poverty by design* [56]. Habitat loss could be linked to degradation due to the cutting down of trees for timber, logging and firewood and deforestation for development, which also affects the aesthetics of the area. Although some drivers of change for cultural services are indirect, the results are nevertheless visible in ecosystems.

When it comes to provisioning ESs, crop production, livestock and freshwater were reported to be the most degraded services that have a direct impact on livelihoods (Table 3). This perception could be linked to the direct benefit that people get from these individual services and their levels of availability to the wider indigenous communities [21]. Furthermore, people reported that they consume and sell crops. Not only do these have added nutritional value but they also help generate income for the sustenance of households [57]. Respondents reported that they reared livestock for both consumption and selling. Moreover, the accessibility of these individual provisioning services to community members is significantly influenced by employment opportunities and financial resources including those gained from informal employment at local scale [58]. Collectively, these factors affect the levels of production and consumption of these provisioning services as well as their sustainability.

Poverty/economic influence, lack of technology, climate change and legislation/politics were reported to be the biggest drivers of change to provisioning ESs. Here, the low overall southern African unemployment rate linked to poverty directly reflects how people are unable to maintain their livelihoods because of unemployment, leading to the perception that provisioning services are degrading [59]. In terms of the lack of technology, respondents reported that there is not enough infrastructure, for example, machinery to drill boreholes, agricultural machinery and domestic water-purifying tools, to support their livelihoods. Additionally, perceptions of legislation and politics could be driven by the notion of corruption and nepotism in the area. For example, it was reported that in Zimbabwe the United Nations's Pfumvudza initiative of 2020 for smallholder farmers only benefits people that bribe officers for seeds and fertilizers in that area [60]. Climatic factors could also be attributed to the perception of degradation in provisioning services. This is explained by the direct disbenefit that people experience in the variations of climatic conditions for their crops, livestock and timber production during growing seasons, leading to poor yield and disease [61]. Additionally, wild fruits and *mopane worms* foraged from the protected area seem to be declining due to the reported climatic conditions. Compared to cultural ESs, provisioning ESs were deemed more degraded. These results suggest that people's perceptions of ESs and their degradation are linked to their daily need for survival rather than the quality of their lives [51] (Appendix 1, Table A6).

Limited agricultural management practices and low soil fertility leading to reduced economic viability and land abandonment were identified as additional causes of the degradation of provisioning ESs related to land use. These are conditions outlined for the management of semi-arid ecosystems through land amelioration, land reclamation and nutrient recycling through crop rotation [62]. Other changes were associated with both natural and anthropogenic factors, mainly the human-wildlife conflict in the area [63]. In this regard, respondents reported that elephants, buffalos, baboons and monkeys from the national parks vandalise and destroy their crops and livestock, leading to their destruction and scarcity [64].

### 4.2 Ecosystem services and sociodemographic factors

Considering sociodemographic factors provides further dimensions to the understanding of the perceptions of ESs’ availability and change. An important finding from the study shows that people from Zimbabwe perceive provisioning ESs to be more degraded than people from South Africa. Additionally, females in both countries perceived both provisioning and cultural services to be degraded (Table 4). It is interesting to note that although only females (and not other genders) perceived provisioning services to be degraded, all genders perceived cultural services to be degrading. This could be attributed to the fact that people of all genders experience similar living conditions that have an impact on how they live their lives in relation to cultural ESs [61].

Additionally, the high unemployment rate for all genders in the South African and Zimbabwean samples was mostly within the age group that is considered the breadwinners. Since the unemployment rate is high, the observation is that no matter who you are and where you come from, people are equally affected, thus altering their perceptions of ESs’ availability, change and degradation. This observation corroborates the results of [5,65,66] who in their studies observed that socio-demographic factors such as gender and nationality influence ESs perceptions in developing countries. Moreover, household size was seen to have an influence on perceptions of provisioning services [5]. observed similar results, stating that households that had more members to support were more likely to perceive ESs to be unavailable or degraded. Sociodemographic factors such as age and education did not have any influence on the perceptions of both provisioning and cultural ESs. This could also be attributed to the fact that the majority of the respondents within the prevalent age group of 55–65 years in the study had only a basic education. This reflects the reality of rural livelihoods where education is not prioritized by households due to financial reasons [67].

### 4.3 Ecosystem services and human wellbeing indicators

ESs play a vital role in HWB and livelihood sustenance through their material and non-material benefits impacting people's quality of life. This consideration is critical for vulnerable indigenous communities as most policy frameworks aimed at ESs' accessibility in these areas are not inclusive [22]. In our study, we noted that indigenous and local people who have lived within the context of a particular ecosystem establish distinct ecosystem services-well-being linkages (Fig. 4). This is seen in people's perceptions of ESs and HWB indicators within a degrading environment. Furthermore, indigenous people are frequently politically marginalized and thus excluded from decisions that affect resources that they have used, and often protected, for long periods of time [68]. Although this is the case for many indigenous communities that are situated around protected areas, our study also acknowledges the complexity of linking ESs and HWB domains in different situations.

Some survey participants reported that individual cultural ESs such as scientific research, tourism and recreation/landscape recreation do not contribute to their wellbeing. Perceptions towards scientific research could be linked to “*research fatigue*” that the communities have been experiencing [24]. Communities' negative perceptions and attitudes towards scientific research could have also been as a result of them not experiencing immediate change after research activities. With regards to tourism, local people reported that they do not directly benefit from tourists that visit either the Kruger national park or the Gonarezhou national park. Instead, they feel exploited by the park management as tourists usually roam their streets without supporting them economically (Appendix 1, Table A9). Furthermore, people believe that they are not benefiting from the parks' tourism, through employment opportunities or other viable economic opportunities. Additionally, as [59] found that people’ perceptions are altered people's due to the political marginalisation that people experience from government and parks management for tangible sustainable solutions towards their livelihoods.

The biggest contributors to HWB within cultural ESs were cultural heritage, traditional knowledge, education and ecological knowledge. These are essential pathways to human health development, through intellectual, cultural and spiritual practices [64]. Cultural practices reflect human-nature relations that emerge through opportunities for recreation [69], creativity [70], producing and caring, or gathering and consuming natural products for physical and mental health [64]. Intellectual practices construct human-nature relations emerging through learning and gaining new knowledge about the benefits of natural resources and how they can be harnessed for physical wellbeing, usually, through indigenous knowledge systems [24]. In our context, indigenous populations thus have extensive knowledge of their local environments, including knowledge of medicinal plants, which are important for medical treatment within local communities and crucial for their futures in light of the potential unprecedented loss of biodiversity that we are facing [22]. Spiritual practices reflect the human-nature relations that emerge through opportunities for spiritual and religious activities, such as rituals and religious activities in sacred natural places; or using plants and animals in ceremonies, for example the *Adansonia digitata* tree for spiritual fulfilment [5].

Perceptions of provisioning ecosystem services were mostly consistent across the indigenous communities; this could be because these services provide direct material benefits to people [64]. For example [71], observed that traditional and agricultural practices in accessing ESs are better adapted to a changing climate as well as help to conserve and restore natural resources. This is consistent with our study as individual provisioning ESs that contribute the most to people's wellbeing and livelihoods were outlined as crop production, livestock and freshwater. These services improve livelihoods directly through consumption and selling, for example, with harvested crops and livestock; whilst abundant water allows for the healthy growth of these ESs. Mental health is often attributed to provisioning services because of the latter's direct impact on HWB. Respondents in this study reported poor mental health and stability and provisioning services such as timber harvesting and fodder & forage as having a negative impact on their mental health. This could be attributed to the land degradation in the area and the lack of employment that diminishes access to these provisioning services, thereby perpetuating poverty in rural areas close to transboundary protected areas.

The link between ESs and human livelihoods is crucial for enhancing human wellbeing, especially for people living in rural areas. Policy and policy makers must acknowledge and strengthen socio-ecological systems and rural stakeholders for effective, sustainable and structured mitigation strategies towards ESs management [54]. observed that whilst policy documents are increasingly taking HWB into account, linking it to nature is still in its infancy, an observation made by this study. Additionally, our study highlights the complexities of linking ESs and HWB domains according to local people's perceptions. In this regard, policy frameworks ought to guide ESs' management decisions in context and in practice, for rural communities by rural communities. Indigenous communities, especially those living in close proximity to remote protected areas, can contribute towards developing unique assessment measures such as ESs-HWB indicators for future sustainable policy-decision-making [14].

## 5 Practical implications for practice and future research

### 5.1 Unemployment and ESs

When taking socio-demographic factors into account in our study, we identified a high rate of unemployment which was not related to other variables such as the level of education of a participant. Employment and economic-related activities are ways in which people can ensure sufficient livelihoods for themselves, their families and their communities at large [58]. This, indeed, would be a factor contributing to the wellbeing of the population [72]. One way to address this is to provide more employment opportunities for the inhabitants of the study area, considering the economic potential from the cultural and provisioning ESs here. Although the parks’ management have tried to create employment in the conservation area [71,73], participants in our study also disclosed that jobs here are offered through nepotism and corrupt practices. The latter could be addressed by ensuring greater transparency in the distribution of provisioning and cultural ESs resources and jobs in the area, for ensuring human wellbeing and, accordingly, promoting a positive perspective towards ESs availability.

### 5.2 Human-wildlife conflict

The participants mentioned that wild animals from the conservation area damaged their agricultural products like crops and livestock, which in turn affects availability of both provisioning and cultural ESs (Appendix 1, Table A8). This observation is not new, as similar human-wildlife conflicts have been recorded in other conservation areas, for example in Kenya [74]. Through biodiversity conservation policy, national parks provide a safe haven for nature, which includes wild animals. However, as observed, wildlife conservation efforts are accompanied by wildlife conflicts with human settlements [75]. This highlights the complexities of issues related to human-wildlife conflict; where only a few resolutions have been explored in similar contexts [76]. Moreover, some international policies to advance the conservation of wildlife in African countries do not consider the safety, activities or interests of inhabitants living close to the conserved areas [77]. Whilst these policies, for example, the Convention on International Trade in Endangered Species of Wild Fauna and Flora (CITES) see opportunities for further environmental conservation and protection of wildlife [78], conflicts from the abundance of wildlife and its impacts on rural livelihoods are somewhat ignored [79]. In this regard, policies must integrate indigenous people's perspectives on wildlife management through further contextual research, for progressive sustainable biodiversity conservation.

### 5.3 Benefits from tourism

Our study found that people in the conservation area do not seem to experience adequate benefits and impacts from tourism activities, associated with cultural ESs. Here, we recommend that more initiatives be implemented for communities to benefit more from revenue generated through tourism. One such commendable initiative is the community development program implemented in the GLTFCA by the Peace Parks Foundation for community development [71]. However, more such programs and policies should be undertaken and implemented. Management authorities of these parks could allocate revenue generated at the parks to funds which could be used to establish needed infrastructure in the community, such as schools, clinics, boreholes, etc. It should be publicized that these developmental projects were financed by revenue generated from the national parks for transparency.

### 5.4 Indigenous people, perceptions, future research and policy-making

It is important that indigenous people's perceptions are considered in ESs-related policy-making [77]. Our and similar studies could be considered as a baseline towards the measurement of local people's perceptions, which should inform policy-making in rural transboundary conservation areas. Further studies could explore perceptions of regulating and supporting ESs and their links to human well-being in this area. Furthermore, field studies must be correlated with robust remotely-sensed ESs assessments, to quantify habitat quality and landscape changes and their linkages to human wellbeing, to progress sustainable conservation in these areas.

### 5.5 Limitations of the study and applicability

The study used a snowball sampling technique to select key informants for interviews. These informants were primarily community leaders who possess in-depth knowledge of the study area and the provisioning and cultural ESs. However, this approach could be considered a limitation, as relying solely on community leaders may introduce bias and limit the diversity of perspectives at large. Future work could involve selecting a broader range of participants [80], including general community members, to serve as key informants. This adjustment would likely reduce the potential for bias and enhance the representativeness of the collected information, providing a more comprehensive and inclusive view of the study. Another potential limitation of the study is that it did not incorporate remotely sensed imagery to directly compare land use and land cover (LULC) changes with local perceptions of provisioning and cultural ecosystem services. Integrating remotely sensed data could provide a more objective assessment of how LULC impacts these ecosystem services, complementing community perceptions.

The methods used in our study are applicable to different scenarios with similar contextual characteristics. A review by [81] showed comparable methodological research conducted in different global regions that can be effectively adapted to diverse areas and contexts. For example, the influence of subjective perceptions on the valuation of green spaces has been assessed in Japan [82], [83] explored the sociocultural influence of ESs domains in European protected areas and [84] investigated how the perceptions of climate change influence ESs and human wellbeing in the United States of America. The studies above share a common methodological structure with our work by utilizing mixed approaches that integrate quantitative data and qualitative insights for different areas and contexts. By aligning people's perceptions with socio-ecological parameters, they demonstrate how diverse local contexts can inform and refine global frameworks, emphasizing the applicability of our methods.

## 6 Conclusion

This study used workshops and questionnaire surveys to explore different perspectives of ESs’ availability, changes, degradation, as well as drivers of their changes. Our study shows that indigenous communities around the Greater Limpopo Transboundary Conservation Area are aware of provisioning and cultural ESs and that they perceive them to be degrading. Quantifying these perceptions has allowed us to link the ESs to human well-being domains and indicators for the sustainability of indigenous people living adjacent to the protected areas. This study is not only a reflection of the contribution that ESs make to the quality of life for these people, but it also sheds light on the resources that local people need for survival that have instead been allocated to the protected areas. As is widely known, local people are aware of the ecological and economic functions of the protected areas. Our results show that they, however, perceive themselves as not benefitting from these functions.

Overall, our study acknowledges that for indigenous people, their connections with natural systems will depend on the status and type of the natural ecosystem, as well as upon the values, knowledge and rights to a particular cultural and provisioning ESs. These communities provided a unique aspect towards bridging the gap between ESs and HWB for improved livelihoods. Finally, applying indigenous perspectives of ESs and their roles in HWB can improve approaches to understanding and implementing contextual, sustainable ESs management within conservation areas, globally.

## 8 English editor

Miss Amina Omar Ismail – University of Johannesburg, aminaoismail@worldonline.co.za.

## CRediT authorship contribution statement

**N.A. Nyathi:** Writing – original draft, Methodology, Funding acquisition, Formal analysis, Data curation, Conceptualization. **W. Musakwa:** Writing – review & editing, Supervision, Methodology, Data curation. **D.M. Azilagbetor:** Writing – review & editing, Validation, Investigation. **N.J. Kuhn:** Writing – review & editing, Supervision, Project administration, Conceptualization.

## 7 Disclosure Statement

No potential conflict of interest was reported by the author(s).

## Data availability Statement

The data that support the findings of this study are available when requested. Data are available at the University of Basel and University of Johannesburg data repository archive.

## Funding

This research was funded by the Swiss Government Excellence Scholarship (ESKAS) grant number 2021.0499. The pilot project was funded by the 10.13039/501100001321National Research Foundation of South Africa (10.13039/501100001321NRF) grant number PR_SFH211220655056.

## Declaration of competing interest

The authors declare the following financial interests/personal relationships which may be considered as potential competing interests: Nesisa Analisa Nyathi reports financial support was provided by Swiss Government Excellence Scholarship (ESKAS). Nesisa Analisa Nyathi reports financial support was provided by 10.13039/501100001321National Research Foundation of South Africa (10.13039/501100001321NRF). If there are other authors, they declare that they have no known competing financial interests or personal relationships that could have appeared to influence the work reported in this paper.

## Footnotes

Appendix A Supplementary data to this article can be found online at https://doi.org/10.1016/j.heliyon.2024.e41448.
