# Supplementary material for: Perceptions of cultural and provisioning ecosystem services and human wellbeing indicators amongst indigenous communities neighbouring the greater limpopo transfrontier conservation area
Source: Heliyon. 2024 Dec 25;11(1):e41448. doi: 10.1016/j.heliyon.2024.e41448 (PMC11751526; doi:10.1016/j.heliyon.2024.e41448)
Supplement: Multimedia component 1 [file mmc1.pdf]

Variable definitions for paper

Study Sites According to District and Municipality

- Chiredzi town council, (Malilangwe, Zimbabwe)
- Chiredzi town council (Malipati, Zimbabwe)
- Vhembe District Municipality (Masisi/Bende Mutale, South Africa)

Table 1 : Village Names: 43 Villages in total.

**Baza ward 5**	**Ben Jokolosi**	**Bende Mutale**	**Chavani**	**Chidhumo ward 5**	**Chidi ward 5**	**Chihosana ward 5**	**Chivhande ward 5**
**Chudu ward 5**	Dovho ward 12	Duluthulwa	Gadzanai ward 5	Gonakudzingwa ward 12	Gonakudzingwa ward 13	Hasani Zambiyani ward 5	Hlomela ward 13
**Jerero 1 ward 5**	Jokolosi ward 5	Lisinga ward 5	Kephas Kostini ward 5	Madangani	Makhese ward 13		Klasiya ward 5
**Kufamuni ward 5**	Kupani Ndali ward 5	Masisi ward 112	Magumbe ward 5	Mahatlani ward 5	Masiya ward 5	Makhubele	Makweza ward 5
**Manhandle ward 13**	Marhanele ward 13	Mubeto ward 5	Masisi ward 12	Masisi ward 13	Mukhuvo ward 13	Mategu ward 5	Matema
**Matengu ward 5**	Mavhuve ward 5	Pahlani Ndali ward 5	Mugumbe ward 5	Mukhurhimbi ward 5	Tshilamusi	Musimeki ward 13	Tshenzhelani ward 12
**Ngwengweja ward 5**	Njitimani ward 5	Panzeka ward 5	Phiri ward 5	Tshikuyu	Welani ward 5	Zinjiva ward 5	

Table 2: Definitions for variables used in the questionnaire.

**Variable**	**Definition**	**Source**
**Socio-Demographics**	-Factors that detail respondent information based on gender (sex), education, age, household size, employment and nativity ("non-native," – refers to people who do not originally come from the two study locations but have relocated there and now reside in these areas)	(Zoeller et al., 2021)
**Cultural Services**	-Non-material benefits that people obtain from nature, including spiritual, aesthetical, educational and recreational values.<br>-These benefits are not tangible and therefore are difficult to assign a monetary value.	(Kosanic & Petzold, 2020)
**Provisioning Services**	-Material goods or benefits that can be harvested and easily quantified and are derived from the eco- systems to be directly used by local people.	(Boafo et al., 2016)
**Human Well Being Indicators**	-Factors influencing an individual's ability to live well regardless of geography, age, culture, religion or political environments	(Jones, 2013)
**Human Wellbeing Domains**	-Holistic view of human well-being including subjective, economic and environmental elements alongside basic human needs<br>-Allows for the effect of changes in (objective and subjective) human well-being as a result of changes in ecosystem services	(Smith et al., 2013)<br>(Rendón et al., 2019)

Table 3a: Definitions for the individual provisioning services that were identified during key interviews.

**Provisioning Services**	**Definition**	**Source**
Livestock	Home reared animals that help sustain livelihoods. Animals such as cattle, goats, pigs, chickens etc.	(Leroy et al., 2024)
Crop	Crops produced in homesteads for subsistence farming. Eg, Maize, okra, beans etc	(Polasky et al., 2011)
Timber	Wood prepared and processed for use in building and carpentry	(Grammatikopoulou & Vačkářová, 2021)
Fodder & Forage	Processed and prepared feed stored for livestock. Eg hay, silage	(Boafo et al., 2016)
Soils	A combination of practices used to protect the soil including top soil for growing crops.	(IPBES, 2019)
Freshwater	Fresh water input from ground water and water received from rains	(Vári et al., 2022)
Traditional Medicine	Medicinal plants used to treat ailments. Normally weeds.	(Boafo et al., 2016)
Wild Plants	The ability for nature to revive for provisions e.g wild fruits	(Boafo et al., 2016)

Table 3b: Definitions for the individual provisioning services that were identified during key interviews.

**Cultural Services**	**Definition**	**Source**
Cultural Heritage	Heritage and cultural identity provided by the landscape	(Tengberg et al., 2012)
Education/Interpretation	Education about the environment and its benefits	(IPBES, 2019)
Ecological Knowledge	Awareness of the benefits of ecological factors of the environment for day to day living	(Wu et al., 2022)
Landscape aesthetics	Sensory cognition of landscape qualities for better livelihood and human health	(Hayat, 2020)
Scientific research	Research carried out for biodiversity conservation	(Musakwa et al., 2020)
Traditional knowledge	Indigenous knowledge systems on the environment and its impact on health, spirituality and sustainability	(Boafo et al., 2016)
Tourism/Ecotourism	Tourism for economic purposes inside and outside the national parks	(Saeed et al., 2022)
Recreation	Recreational activities carried out by people for fun and relaxation and a peace of mind	(Thiemann et al., 2022)

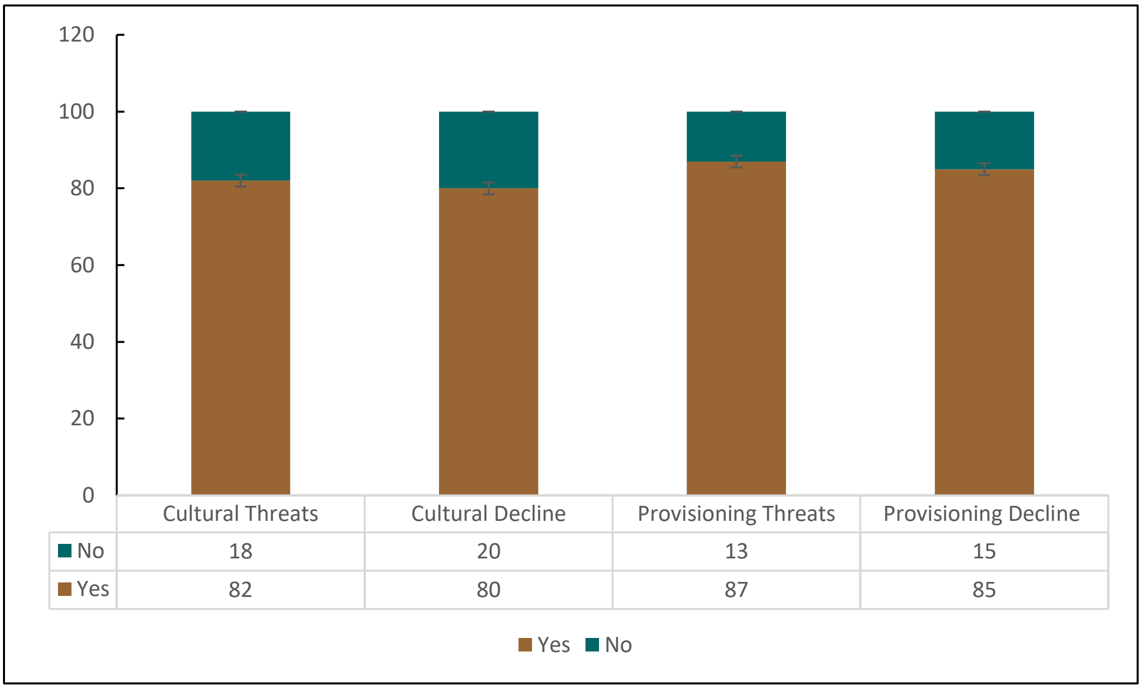

Figure 1: distribution of the amount of people that acknowledged ES degradation and threats for both cultural and provisioning services**.**

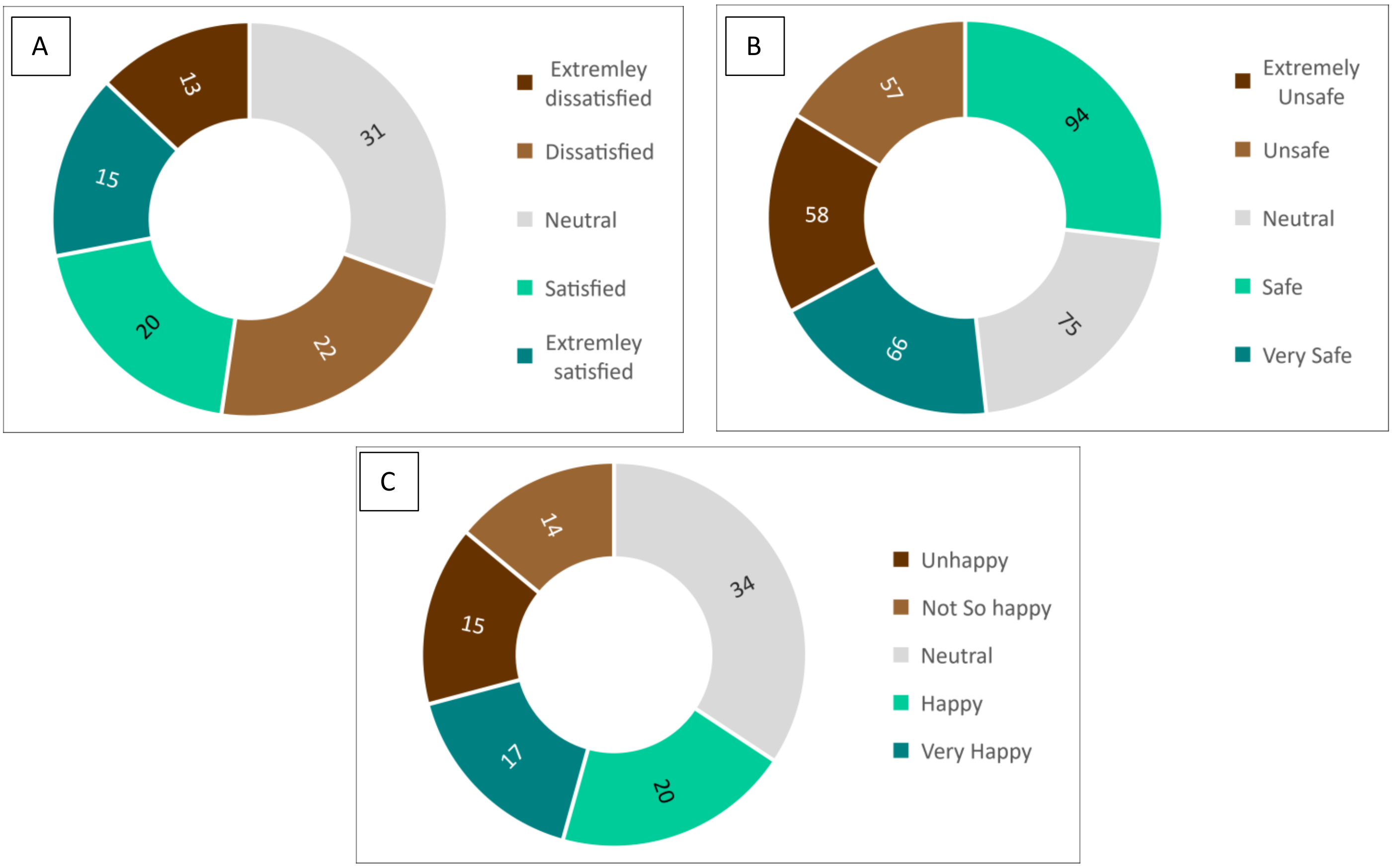

Figure 2: distribution of the amount of people that (a) are satisfied with their lives, (b) feel safe where they live and (c) are overall happy.

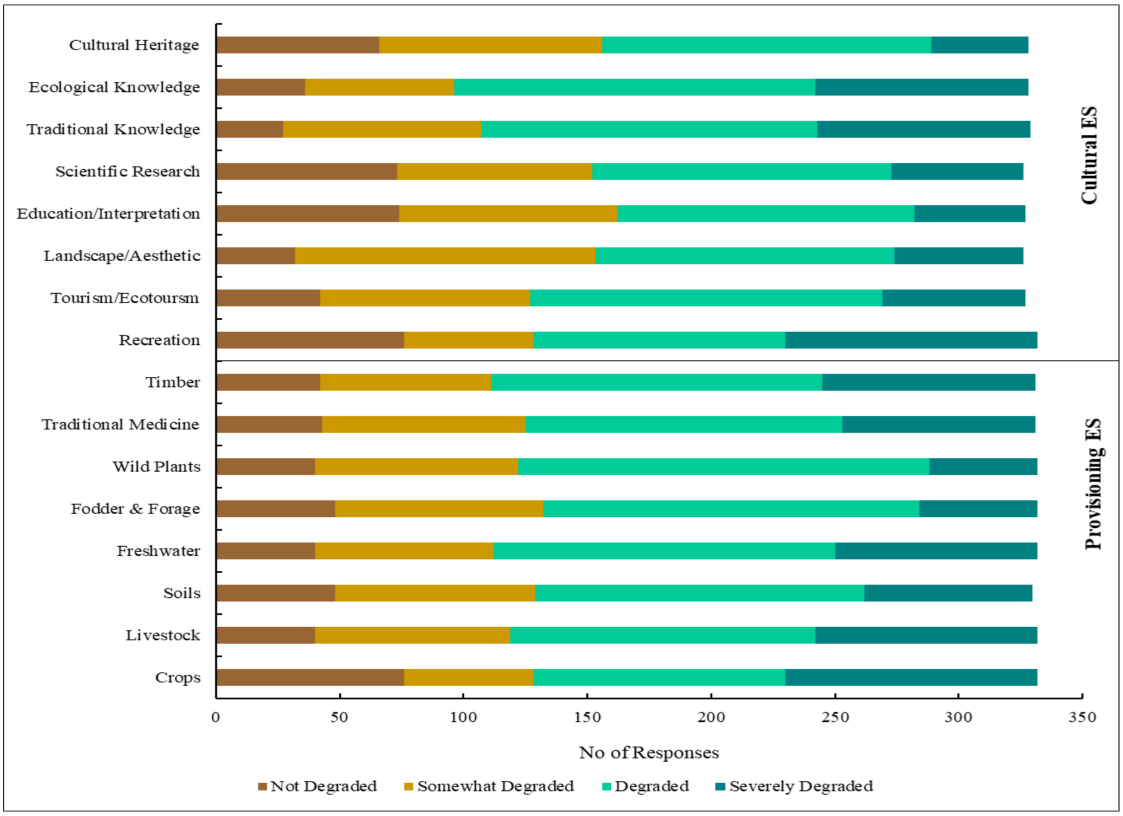

Figure 3: Perceptions of ES degradation for both cultural and provisioning services

Table 4: Key Interviews on drivers of ESs changes. Here we present some key statements from the respondents. More are available upon request

**Key Informant**	**Drivers of Change**	**Some Key Statements***
1	Poverty/Economic Influence	"All the time, it is difficult to feed everyone in the house especially when no one is working"
2	Lack of Technology	"We do not have the technology to drill boreholes or fertilisers for us to access freshwater or to take care of our environment"
3	Legislation/Politics	"When there are some political elections, we face challenges when it comes to accessing food"
4	Habitat Loss/Degradation	"Usually, animals like baboons destroy our fields and crops, so it makes it impossible to access food sometimes"
5	Climate Change	"We face a lot of drought and climate related pressure on our provisioning services. The trees are not growing as tall and there are not so much wild fruits to forage"
6	Cultural activities	"Sometimes, we have to perform cultural activities with our livestock such as cows, sheep or chickens. Although this is good for our spiritual fulfilment, our livestock is depleting because of this. We cannot buy more because we are unemployed"

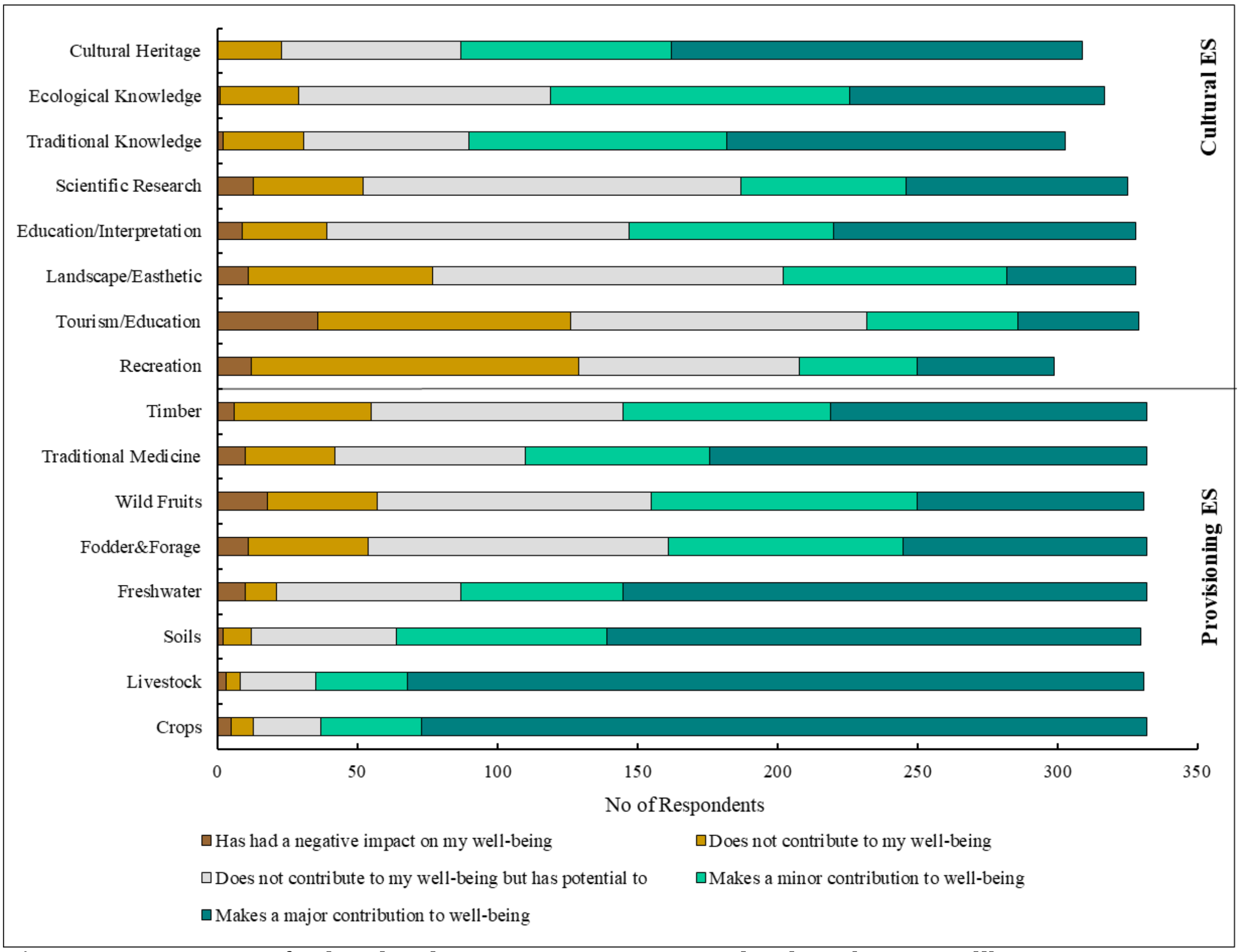

Figure 4: Perceptions of cultural and Provisioning ESs impact levels on human wellbe

	Selling_n	Consumption	ES Category	Other Uses
**Crops**				
**Maize (Umbila/Nsima)**	47	53	Provisioning	
**Cabbage**	44	56	Provisioning	
**Okra**	60	40	Provisioning	
**Onions**	60	40	Provisioning	
**String Beans**	72	28	Provisioning	
**Sugar Cane**	80	20	Provisioning	
**Sorghum**	84	16	Provisioning	
**Watermelons**	50	50	Provisioning	
**Kale**	20	80	Provisioning	
**Marula**	100	0	Provisioning, Cultural	Processed into alcoholic beverages for economic use
**Bananas**	50	50	Provisioning, Cultural	Leaves used as preservatives to store food, used to feed livestock
**Smellyberry fingerleaf fruit (Tsubvu)**	60	80	Provisioning, Cultural	Used as timber for logging, Aesthetics
**Monkey Orange**	20	80	Provisioning, Cultural	Treating Snake bites and wood for timber
**Bird Plum (Nyii)**	65	35	Provisioning, Cultural	Roots soaked in water and used for stomach/abdominal pains
**Tomatoes**	50	50	Provisioning	
**Avocados**	50	50	Provisioning, Cultural	Seeds used to extract oil, leaves used to treat stomach diseases, bark used for firewood
**Macademia nuts**	100	0	Provisioning, Cultural	Aesthetics
**Litchis**	80	20	Provisioning, Cultural	Aesthetics, medicinal purposes
**Bell Peppers**	90	10	Provisioning	
**Snot apples (uXakuxaku/morojwa)**	20	80	Provisioning, Cultural	Timber used to make domestic items such as spoons, improves the body's immune system, leaves used as manure
**Wild medlar (Umviyo/Munjiro)**	20	80	Provisioning, Cultural	Used to make beer for economic purposes

**Baobab Fruit**	70	30	Provisioning, Cultural	Seeds used to exctract oil, Tree reserved as sacred for religeous purposes
**Red Milkwood (Nhlantswa/Mubululu)**	80	20	Provisioning, Cultural	Alleviating Vitamic C deficiency
**Wild Plum (Mothekele/Umgwenya)**	60	40	Provisioning, Cultural	Bark is used for traditional medicine for treating skin diseases, Timber use
**Livestock**				
**Cattle**	50	50	Provisioning	
**Goats**	50	50	Provisioning, Cultural	Traditional Practises
**Sheep**	80	20	Provisioning, Cultural	Traditional Practises
**Pigs**	70	30	Provisioning	
**Chickens**	40	60	Provisioning	
**Quails**	80	20	Provisioning	

	Selling_n	Consumption	ES Category	Other Uses
**Crops**				
**Maize (Umbila/Nsima)**	47	53	Provisioning	
**Cabbage**	44	56	Provisioning	
**Okra**	60	40	Provisioning	
**Onions**	60	40	Provisioning	
**String Beans**	72	28	Provisioning	
**Sugar Cane**	80	20	Provisioning	
**Sorghum**	84	16	Provisioning	
**Watermelons**	50	50	Provisioning	
**Kale**	20	80	Provisioning	
**Marula**	100	0	Provisioning, Cultural	Processed into alcoholic beverages for economic use
**Bananas**	50	50	Provisioning, Cultural	Leaves used as preservatives to store food, used to feed livestock
**Smellyberry fingerleaf fruit (Tsubvu)**	60	80	Provisioning, Cultural	Used as timber for logging, Aesthetics
**Monkey Orange**	20	80	Provisioning, Cultural	Treating Snake bites and wood for timber
**Bird Plum (Nyii)**	65	35	Provisioning, Cultural	Roots soaked in water and used for stomach/abdominal pains
**Tomatoes**	50	50	Provisioning	
**Avocados**	50	50	Provisioning, Cultural	Seeds used to extract oil, leaves used to treat stomach diseases, bark used for firewood
**Macademia nuts**	100	0	Provisioning, Cultural	Aesthetics
**Litchis**	80	20	Provisioning, Cultural	Aesthetics, medicinal purposes
**Bell Peppers**	90	10	Provisioning	

**Snot apples (uXakuxaku/morojwa)**	20	80	Provisioning, Cultural	Timber used to make domestic items such as spoons, improves the body's immune system, leaves used as manure
**Wild medlar (Umviyo/Munjiro)**	20	80	Provisioning, Cultural	Used to make beer for economic purposes
**Baobab Fruit**	70	30	Provisioning, Cultural	Seeds used to exctract oil, Tree reserved as sacred for religeous purposes
**Red Milkwood (Nhlantswa/Mubululu)**	80	20	Provisioning, Cultural	Alleviating Vitamic C deficiency
**Wild Plum (Mothekele/Umgwenya)**	60	40	Provisioning, Cultural	Bark is used for traditional medicine for treating skin diseases, Timber use
**Livestock**				
**Cattle**	50	50	Provisioning	
**Goats**	50	50	Provisioning, Cultural	Traditional Practises
**Sheep**	80	20	Provisioning, Cultural	Traditional Practises
**Pigs**	70	30	Provisioning	
**Chickens**	40	60	Provisioning	
**Quails**	80	20	Provisioning	

Table 5: Indigenous vegetables, fruits, trees and crops reported by respondents as their source of livelihood in the area. Other indigenous sources have multiple uses, including medicinal ones.

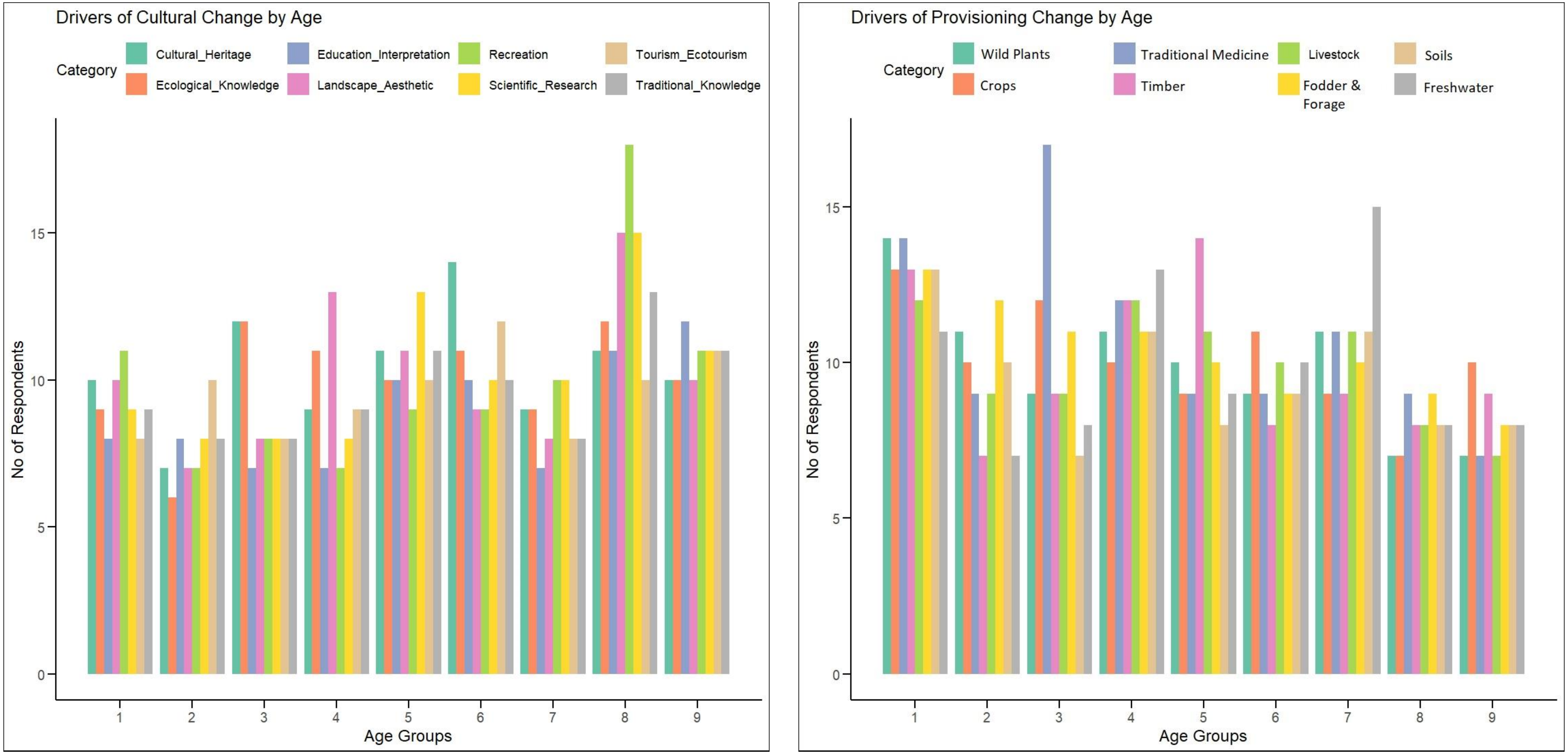

Figure 3: Categorised drivers of cultural ecosystem services by age. Y-axis shows the total number of respondents for both countries and X-axis shows the different age groups with 1 = 18 – 25, 2 = 26 – 32, 3 = 33 – 37, 4 = 38 – 44, 5 = 45 – 49, 6 = 50 – 54, 7 = 55 – 59, 8 = 60 – 65 and 9 = 65 and above.

		Coeff.	Std.err.	t	p	R^2
**Country**	Constant	1.8616	0.092486	20.129	6.06E-60	
	Crops	-0.024973	0.021459	-1.1638	0.24532	0.029393
	Livestock	-0.022992	0.019022	-1.2087	0.22761	0.030473
	Soils	-0.011639	0.019791	-0.58812	0.55684	0.010919
	Freshwater	0.034229	0.020611	1.6607	0.097687	0.0013221
	Fodder & Forage	-0.021166	0.019635	-1.078	0.2818	0.018109
	Fodder & Forage	-0.043674	0.018666	-2.3398	0.019871	0.042764
	Traditional Medicine	-0.02931	0.019601	-1.4954	0.13575	0.031454
	Timber	0.020764	0.020993	0.98912	0.32331	0.0093989
**Gender**	Constant	1.675	0.10776	15.544	1.45E-41	
	Crops	0.030579	0.025002	1.2231	0.22214	6.83E-07
	Livestock	-0.041819	0.022163	-1.8869	0.060024	0.0073541
	Soils	0.024064	0.023058	1.0436	0.29741	0.00052726
	Freshwater	-0.032543	0.024014	-1.3551	0.17627	0.0065689
		-0.015789	0.022877	-0.69015	0.49057	0.0023927
	Wild Plants	0.029479	0.021748	1.3555	0.17615	0.00015285
	Traditional Medicine	0.0096226	0.022837	0.42136	0.67376	0.00047657
	Timber	-0.031681	0.024459	-1.2952	0.19611	0.0070679
**Age**	Constant	3.7169	0.4393	8.461	7.92E-16	
	Crops	0.14777	0.10193	1.4498	0.14804	0.0032968
	Livestock	-0.084622	0.090354	-0.93656	0.34965	2.09E-05
	Soils	0.05295	0.094005	0.56328	0.57362	0.0019112
	Freshwater	0.18511	0.097902	1.8908	0.059501	0.0053744

	Fodder & Forage	-0.13501	0.093266	-1.4476	0.14865	0.00093552
	Wild Plants	-0.21091	0.088661	-2.3789	0.017915	0.0093418
	Traditional Medicine	0.08281	0.093102	0.88945	0.37439	0.0011772
	Timber	0.046091	0.099715	0.46223	0.64421	0.00070594
**Household Size**	Constant	4.6583	0.2252	20.685	3.62E-62	
	Crops	0.0061768	0.052251	0.11821	0.90597	0.00044664
	Livestock	-0.026807	0.046318	-0.57875	0.56314	0.00050466
	Soils	-0.036686	0.04819	-0.76129	0.44701	0.00091237
	Freshwater	0.15019	0.050187	2.9925	0.0029685	0.01597
	Fodder & Forage	-0.10772	0.047811	-2.253	0.024895	0.00048486
	Wild Plants	-0.04003	0.04545	-0.88075	0.37907	0.00019682
	Traditional Medicine	0.078984	0.047727	1.6549	0.098863	0.016607
	Timber	0.084077	0.051117	1.6448	0.10093	0.01845
**Education Level**	Constant	1.862	0.24286	7.667	1.85E-13	
	Crops	0.081102	0.056349	1.4393	0.15099	0.0079457
	Livestock	-0.036342	0.049951	-0.72755	0.46739	0.0011164
	Soils	0.0276	0.051969	0.53109	0.59571	0.005428
	Freshwater	0.040853	0.054123	0.7548	0.45089	0.0066437
	Fodder & Forage	-0.017267	0.051561	-0.33488	0.73792	0.002859
	Wild Plants	-0.0035781	0.049015	-0.073	0.94185	0.0016323
	Traditional Medicine	-0.0067532	0.05147	-0.13121	0.89569	0.0021494
	Timber	0.041248	0.055126	0.74825	0.45482	0.0051314
**Employment**	Constant	2.6219	0.12316	21.288	1.43E-64	
	Crops	0.045218	0.028577	1.5824	0.11449	0.00054412
	Livestock	-0.060386	0.025332	-2.3838	0.017682	0.0069367
	Soils	0.053858	0.026355	2.0435	0.041768	0.0071755
	Freshwater	0.0038972	0.027448	0.14199	0.88717	0.0021609

	Fodder & Forage	-0.018945	0.026148	-0.72452	0.46924	0.00052967
	Wild Plants	0.021733	0.024857	0.87431	0.38257	0.00067482
	Traditional Medicine	-0.0018621	0.026102	-0.071339	0.94317	0.00061624
	Timber	-0.034824	0.027956	-1.2456	0.21375	0.0042316

Table 6: Multi regression analysis for individual provisioning ecosystem services and Socio demographic factors**.**

		**Coeff.**	**Std.err.**	**t**	**p**	**R^2**
**Country**	Constant	1.4194	0.10765	13.186	2.29E-32	
	Recreation	0.0021406	0.020016	0.10695	0.91489	0.00053804
	Tourism/Ecotourism	0.0038653	0.022071	0.17513	0.86108	0.00071488
	Landscape/Aesthetic	0.01512	0.020961	0.72131	0.47121	0.0033124
	Education/Interpretation	0.013986	0.021555	0.64885	0.51687	0.0035567
	Scientific Research	0.017235	0.021393	0.80564	0.42101	0.0021741
	Traditional Knowledge	-0.053234	0.02049	-2.5981	0.0097821	0.0091463
	Ecological Knowledge	0.016479	0.021994	0.74927	0.45421	9.41E-05
	Cultural Heritage	0.0086114	0.020588	0.41827	0.67601	0.0012166
**Gender**	Constant	1.745	0.1229	14.198	2.85E-36	
	Recreation	0.01712	0.022852	0.74918	0.45427	8.10E-05
	Tourism/Ecotourism	-0.011426	0.025199	-0.45341	0.65054	0.0029629
	Landscape/Aesthetic	-0.0034316	0.023932	-0.14339	0.88607	0.0022491
	Education/Interpretation	-0.01083	0.024609	-0.44009	0.66015	0.0045852
	Scientific Research	-0.026392	0.024425	-1.0806	0.28065	0.0076339
	Traditional Knowledge	-0.0055033	0.023394	-0.23525	0.81416	0.0037008
	Ecological Knowledge	-0.011522	0.025111	-0.45885	0.64664	0.0041627
	Cultural Heritage	0.0059114	0.023506	0.25149	0.80159	0.00090076
**Age**	Constant	4.2291	0.50305	8.4069	1.16E-15	
	Recreation	0.10856	0.093535	1.1606	0.24661	0.0019231

	Tourism/Ecotourism	-0.052416	0.10314	-0.5082	0.61164	0.0014351
	Landscape/Aesthetic	-0.10687	0.097953	-1.091	0.27602	0.0054916
	Education/Interpretation	-0.065553	0.10073	-0.6508	0.51561	0.003088
	Scientific Research	-0.025079	0.099971	-0.25086	0.80207	0.00043164
	Traditional Knowledge	0.099795	0.095751	1.0422	0.29804	0.0017053
	Ecological Knowledge	0.030194	0.10278	0.29377	0.76911	0.00027102
	Cultural Heritage	-0.041301	0.096211	-0.42928	0.66799	0.0007896
**Household Size**	Constant	4.9494	0.25886	19.12	6.78E-56	
	Recreation	0.02488	0.048131	0.51692	0.60555	0.00052785
	Tourism/Ecotourism	-0.050426	0.053075	-0.9501	0.34274	0.0010779
	Landscape/Aesthetic	-0.0029248	0.050405	-0.058026	0.95376	4.01E-05
	Education/Interpretation	0.090529	0.051832	1.7466	0.081608	0.0062215
	Scientific Research	-0.019894	0.051443	-0.38672	0.6992	4.99E-05
	Traditional Knowledge	-0.090949	0.049272	-1.8459	0.06578	0.0075316
	Ecological Knowledge	0.0050739	0.052888	0.095937	0.92363	0.00017579
	Cultural Heritage	0.063272	0.049508	1.278	0.20212	0.0041616
**Education Level**	Constant	2.3923	0.27611	8.6644	1.85E-16	
	Recreation	-0.04742	0.051337	-0.92369	0.3563	0.0021008
	Tourism/Ecotourism	0.010492	0.05661	0.18534	0.85308	1.03E-05
	Landscape/Aesthetic	-0.026709	0.053762	-0.49679	0.61966	0.00030779
	Education/Interpretation	0.025752	0.055285	0.46581	0.64165	0.00043675
	Scientific Research	-0.0094524	0.05487	-0.17227	0.86333	7.63E-05
	Traditional Knowledge	-0.0085927	0.052554	-0.1635	0.87022	0.00010518
	Ecological Knowledge	-0.019178	0.056411	-0.33996	0.7341	0.00012032
	Cultural Heritage	0.05538	0.052806	1.0487	0.29504	0.0019516
**Employment**	Constant	2.6029	0.13961	18.644	5.54E-54	

	Recreation	0.0061015	0.025958	0.23505	0.81431	3.50E-05
	Tourism/Ecotourism	-0.03574	0.028625	-1.2486	0.21268	0.0002797
	Landscape/Aesthetic	0.013619	0.027185	0.50097	0.61672	0.0014132
	Education/Interpretation	0.086749	0.027954	3.1032	0.0020742	0.018356
	Scientific Research	-0.020542	0.027745	-0.74041	0.45956	2.02E-05
	Traditional Knowledge	0.0013401	0.026574	0.050429	0.95981	6.27E-05
	Ecological Knowledge	-0.011959	0.028524	-0.41926	0.67529	0.00020237
	Cultural Heritage	-0.027182	0.026701	-1.018	0.3094	0.00069415

Table 7: Multi regression analysis for individual cultural ecosystem services and Socio demographic factors.

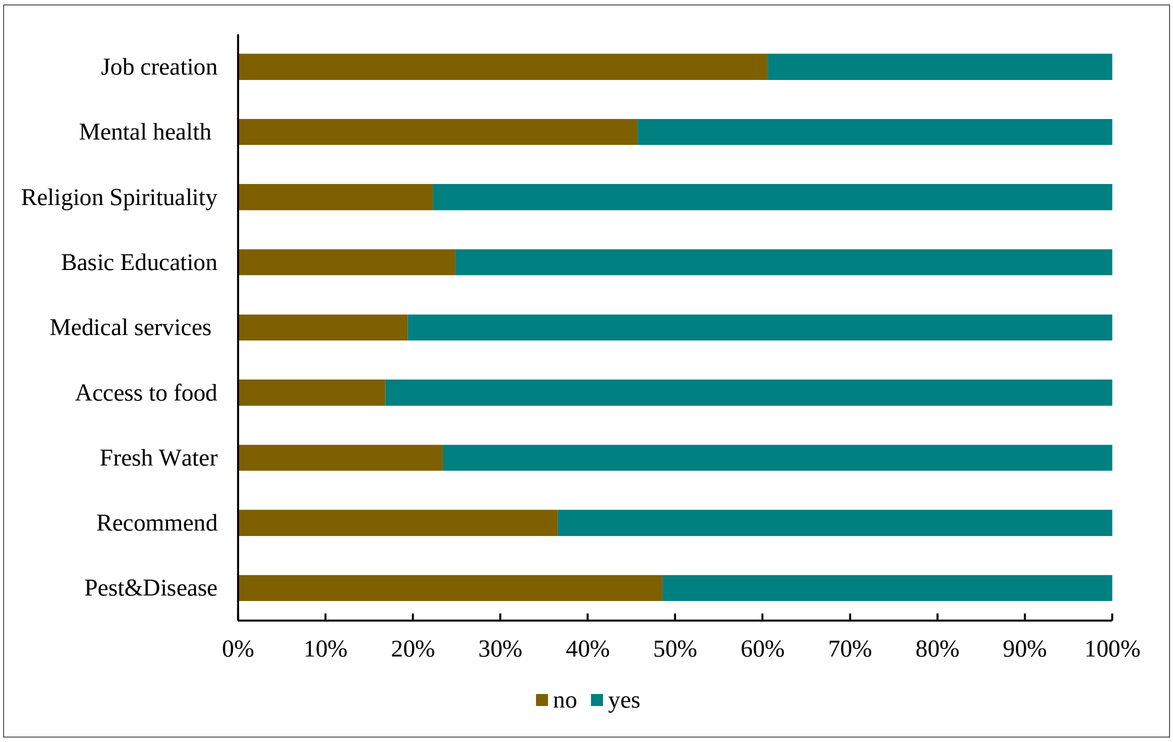

Figure 4: percentage of respondents acknowledging to have access to different human well-being indicators in both countries.

Table 8: Statements from respondents that formed the human well-being domains established by MAXQDA.

Health	Social Cohesion	Spiritual and cultural fulfillment	Connection to nature	Satefy and security	Living standards	Life satisfaction
assistance with sanitizers and PPE especially for the pandemic now. We are not allowed to harvest indegenous plants in the park that will help with remedies to heal ourselves and our children. We get shot. But that is our ancestral lands	My siblings cannot go to school because of the fees, and my parents could not go to work because of old age and we depended on hardship allowance. This makes it hard on us, but maily, we cannot meet opther people to socialise	Sometimes we are banned from harvesting wild fruits to brew the traditiol beer to sell of which is my other source of income and to make rituals for my familys safety	Usually, animals like baboons destroy our fields and crops, so it makes it impossible to access food sometimes	People here steal livestock, crops. I do not feel safe most of the times.	I dont have farming equipments, so its so difficult for me to grow crops which survive any time of weather	We had cattle rears and homestead helpers. We would also like to point out that the government should provide some level of aid to s since 99% of the country is self employed and the informal sector was not allowed to trade during covid
Always being at home changed the dymics of our family in terms of food, mental health and getting access to markets for trading, this was really tough	No longer playing social soccer and travelling to relatives because of lack of income	Attending church is sometimes hard as I cannot bring anything to share with people in terms of food or livestock	Elephants destroy the fields and during drought years	really long distances to be able to access their primary schools. Some children get raped, stolen from and end up either sick or not going to school	Climate change has really changed our ways of liiving. Rain has become scarce, food is not easy to grow or buy, theres major drought during summer and we experience very bad heat waves that kill people	I am not satisfied with my life, I have four children that I have had to marry to older men to receive money from theor 'lobola'. This is how we make a living here, we look our for each other
Covid times were really tough on us, mentally, spiritually and financially. We were expected to wear PPE , walk to the markets and also practice social distancing. This did not work at all and many people died. Also because of lack of education about the virus, lack of vaccines and misinformation about indegenous plants (Zumbani)	I felt stuck at home most of the times as we were not allowed to socialize during COVID-19 so my mental health was affected and to make matters worse, there are no jobs	We often attend church under a tree, but now we cannot because people are cutting down treets for firewood. Atleast we still have the baobab to connect ith our ancestors	I do not feel connected to nature at all.	This is really the worst times to live in this area, we starve and people are mostly dying from hunger than COVID, this doesn't make me feel safe at all	Everything is just expensive, we cannot afford anything, we barely have jobs to even pay rent	Sometimes I look at my life and I regret being born. We are so poor here. The government doesn't care about us. We have so much nature but no one cares. We even have a park right next to us, they make so much money but community members barely benefit anything from that. Those are also our
lack of peace of mind due to no money and no employment, this really affects my mental and physical health. The parks management employ famalies, and so if you don't have a connection, you cannot get a job. My health is declining because I cannot afford medicine for my hypertension	Most of our Children left for South Africa to find greener pastures. Sometines they come home to enjoy the natiure and have a peace of mind from noisy Johannesburg. But this also comes at a cost for us. We are old and have no one to socialise with	My home allows me to do rituals and pray to my ancestors for my spiritual fullfilment. I am able to brew beer and pray for good fortune or good rains	I have a claypot business. I collect wild clay to sell throughout the community to make a living. I feel connected to nature this way	Sometimes baboons come from the park and eat all our crops ad steal livestock from us. We often don't feel safe from them	We only are able to have breakfast everyday	I do not have a job, I don't have money or an education but I am alive. I am grateful to God. I am able to be here, talking to you
Health and food stuffs are very expensive and difficult to access, plus, we often have to walk long distances to access even a clinic. One child died on the way to the hospital because there was no transport to take her to the doctor, also, the roads made it hard to access the route	We don't really have social activities here, people normaly just go to the tarven to drink until their drunk				Its now hard for us to earn a normal living because its now very expensive for us to send our children to school	

Table 9: Benefits of HWB against Human wellbeing Domains**.**

**Ecosystem Services Benefits**	**Well-Being Domain**
Climate, pollution and disease	Connection to nature, health and wellbeing
Reduced hazard risk	Safety and security, health and wellbeing
Farmed Food	Life satisfaction and happiness, health and wellbeing
Reared Livestock	Life satisfaction and happiness, health and wellbeing
Recreation, Tourism	Life satisfaction and happiness, social cohesion, living standards
Wild Food	Life satisfaction and happiness
Fodder & Forage	Spiritual and cultural fulfillment, social cohesion, living standards
Freshwater, soil and sand conservation	health and wellbeing
Research, Education, traditional and ecological knowledge	Life satisfaction and happiness, health and wellbeing, living standards

References

Boafo, Y. A., Saito, O., Jasaw, G. S., Otsuki, K., & Takeuchi, K. (2016). Provisioning ecosystem services-sharing as a coping and adaptation strategy among rural communities in Ghana's semi-arid ecosystem. *Ecosystem Services*, *19*, 92–102. https://doi.org/10.1016/j.ecoser.2016.05.002

Grammatikopoulou, I., & Vačkářová, D. (2021). The value of forest ecosystem services: A meta-analysis at the European scale and application to national ecosystem accounting. *Ecosystem Services, 48*. https://doi.org/10.1016/j.ecoser.2021.101262

Hayat, S. (2020). Ecosystem Services And Human Well-Being. *Inclusive Development and Multilevel Transboundary Water Governance*, 43–59. https://doi.org/10.1201/9781003048688-4

IPBES. (2019). Summary for policymakers of the global assessment report on biodiversity and ecosystem services. In *Intergovernmental Science-Policy Platform on Biodiversity and Ecosystem Services* (Vol. 45, Issue 3). https://zenodo.org/record/3553579#.YfmYTerMI2w

Jones, C. (n.d.). *Cover photo : Children queue up for their daily meal at the Girl Child Network Project , at Kawangware School in Nairobi , Kenya in March 2009 . Photo : Kate Holt / Africa Practice via AusAID Flickr site*.

Kosanic, A., & Petzold, J. (n.d.). A systematic review of cultural ecosystem services and human wellbeing. *Ecosystem Services*, *45*, 101168.

Leroy, G., Boettcher, P., Joly, F., Looft, C., & Baumung, R. (2024). Multifunctionality and provision of ecosystem services by livestock species and breeds at global level. *Animal*, *18*(1), 101048. https://doi.org/10.1016/j.animal.2023.101048

Musakwa, W., Wang, S., Wei, F., Malapane, O. L., Thomas, M. M., Mavengahama, S., Zeng, H., Wu, B., Zhao, W., Nyathi, N. A., Mashimbye, Z. E., Poona, N., Chakwizira, J., Gumbo, T., Mokoena, B., Kaitano, F., Fundisi, E., & Yeni-Letsoko, V. (2020). Survey of community livelihoods and landscape change along the Nzhelele and Levuvhu river catchments in limpopo province, South Africa. *Land, 9*(3). https://doi.org/10.3390/land9030091

Polasky, S., Nelson, E., Pennington, D., & Johnson, K. A. (2011). The impact of land-use change on ecosystem services, biodiversity and returns to landowners: A case study in the state of Minnesota. *Environmental and Resource Economics*, *48*(2), 219–242. https://doi.org/10.1007/s10640-010-9407-0

Rendón, O. R., Garbutt, A., Skov, M., Möller, I., Alexander, M., Ballinger, R., Wyles, K., Smith, G., McKinley, E., Griffin, J., Thomas, M., Davidson, K., Pagès, J. F., Read, S., & Beaumont, N. (2019). A framework linking ecosystem services and human well-being: Saltmarsh as a case study. *People and Nature, 1*(4), 486–496. https://doi.org/10.1002/pan3.10050

Saeed, U., Arshad, M., Hayat, S., Morelli, T. L., & Ali Nawaz, M. (2022). Analysis of provisioning ecosystem services and perceptions of climate change for indigenous communities in the Western Himalayan Gurez Valley, Pakistan. *Ecosystem Services*, *56*(June 2021), 101453. https://doi.org/10.1016/j.ecoser.2022.101453

Smith, L. M., Case, J. L., Smith, H. M., Harwell, L. C., & Summers, J. K. (2013). Relating ecoystem services to domains of human well-being: Foundation for a U.S. index. *Ecological Indicators*, *28*, 79–90. https://doi.org/10.1016/j.ecolind.2012.02.032

Tengberg, A., Fredholm, S., Eliasson, I., Knez, I., Saltzman, K., & Wetterberg, O. (2012). Cultural ecosystem services provided by landscapes: Assessment of heritage values and identity. *Ecosystem Services*, *2*, 14–26. https://doi.org/10.1016/j.ecoser.2012.07.006

Thiemann, M., Riebl, R., Haensel, M., Schmitt, T. M., Steinbauer, M. J., Landwehr, T., Fricke, U., Redlich, S., & Koellner, T. (2022). Perceptions of ecosystem services: Comparing socio-cultural and environmental influences. *PLoS ONE*, *17*(10 October), 1–20. https://doi.org/10.1371/journal.pone.0276432

Vári, Á., Podschun, S. A., Erős, T., Hein, T., Pataki, B., Iojă, I. C., Adamescu, C. M., Gerhardt, A., Gruber, T., Dedić, A., Ćirić, M., Gavrilović, B., & Báldi, A. (2022). Freshwater systems and ecosystem services: Challenges and chances for cross-fertilization of disciplines. *Ambio*, *51*(1), 135–151. https://doi.org/10.1007/s13280-021-01556-4

Wu, B., Liang, W., Wang, J., & Cui, D. (2022). Rural Residents' Perceptions of Ecosystem Services: A Study from Three Topographic Areas in Shandong Province, China. *Land*, *11*(7). https://doi.org/10.3390/land11071034

Zoeller, K. C., Gurney, G. G., Marshall, N., & Cumming, G. S. (2021). The role of socio-demographic characteristics in mediating relationships between people and nature. *Ecology and Society*, *26*(3). https://doi.org/10.5751/ES-12664-260320
