# Supplementary material for: Perceptions of cultural and provisioning ecosystem services and human wellbeing indicators amongst indigenous communities neighbouring the greater limpopo transfrontier conservation area
Source: Heliyon. 2024 Dec 25;11(1):e41448. doi: 10.1016/j.heliyon.2024.e41448 (PMC11751526; doi:10.1016/j.heliyon.2024.e41448)
Supplement: Multimedia component 2 [file mmc2.pdf]

Ecosystem Services (ES) in the Greater Limpopo Trans-frontier Conservation Area Questionnaire

Coded Document

1. Please tick the box to confirm that you consent to participating in this research *

   Mark only one oval.

   1 - Yes

   2 - No

Biography

2. Location: Village Name *
3. Gender *

   Mark only one oval.

   1 - Male

   2 - Female

   3 -Prefer not to say

4. Age *

   Mark only one oval.

   1 - (19 – 25)

   2 - (26 – 32)

   3 - (33 – 37)

   4 - (38-44)

   5 - (45 – 49)

   6 - (50 -54)

   7 - (55 – 59)

   8 - (60 – 65)

   9 - (65 and above)

5. Household Size *

Tick all that apply.

1 - 1
2 - 2
3 - 3
4 - 4
5 - 5
6 - Above 5

6. Education Level *

Mark only one oval.

1 - Primary Education
2 - High School
3 - College level
4 - University Level
5 - No Education
6 - Other

7. Employment *

Tick all that apply.

1 - Employed
2 - Self-Employed
3 - Unemployed

8. Is the area around Kruger National Park your indigenous homeland?

Mark only one oval.

1 - Yes
2 - No
3 - I don't Know

9. Is the area around Gonarezhou National Park your indigenous homeland?

Mark only one oval.

1 - Yes
2 - No
3 - I don't Know

Cultural ESS

*Recreation *Tourism and Ecotourism *Landscape/aesthetic beauty *Education/ interpretation *Scientific research *Traditional *Ecological Knowledge *Cultural heritage

10. Do you believe the cultural services listed above have declined? *

Mark only one oval.

1 - Yes

2 - No

11. Do you think the cultural services listed above are threatened in the area?*

Mark only one oval.

1 - Yes

2 - No

12. Overall, what do you think are the drivers of change with regards to cultural* services in your area (please tick all that apply).

Tick all that apply.

1 - Climate Change

2 - Legislation/Policy
3 - Agricultural Activities
4 - Values/Mentality/Attitudes
5 - Poverty/Market Economy
6 - Lack of Technology
7 - Urbanisation
8 - Corruption

Provisioning ESS

*1. Crop Production, *2. Livestock, *3. Soil Conservation, *4.Water Yield, *5.Sand fixation, *6.Carbon Sequestration, *7.Habitat, *8Nature, *9Landscape Recreation

13. Do you believe the provisioning services listed above have declined?

Mark only one oval.

1 - Yes

2 - No

14. Do you think the Provisional services listed above are threatened in the area? *

Mark only one oval.

1 - Yes

2 - No

15. Overall, what do you think are the drivers of change with regards to provisional* services in your area (Please tick all that apply).

Tick all that apply.

1 - Climate Change
2 - Legislation/Policy
3 - Agricultural Activities
4 - Values/Mentality/attitudes
5 - Poverty/Market Economy
6 - Lack of Technology
7 - Urbanization
8 - Corruption

Basic Human Needs & Human Wellbeing

16. Does the area help mitigate pest and disease? *

Mark only one oval.

1 - Yes

2 - No

17. Would you recommend your area of living to someone else? *

Mark only one oval.

1 - Yes

2 - No

18. Is the area that you live at considered safe? With 1 being extremely unsafe and 5 extremely safe *

Mark only one oval.

	1	2	3	4	5	
Ext U	◯	◯	◯	◯	◯	Ext Safe

19. Do you have access to fresh water? *

Mark only one oval.

1 - Yes

2 - No

20. Do you have access to food in general? *

Mark only one oval.

1 - Yes

2 - No

21. When do you face challenges when it comes to accessing food?

22. Do you have access to medical services in your area? *

Mark only one oval.

1 - Yes

2 - No

23. Do you have access to basic education in your area? *

Mark only one oval.

1 - Yes

2 - No

24. All things considered, how satisfied are you with your life as a whole? *

Mark only one oval.

	1	2	3	4	5	
V. Di	◯	◯	◯	◯	◯	V. Satisfied

25. On a scale of 1-5 how happy are you, in general? *

Mark only one oval.

	1	2	3	4	5	
Unh	◯	◯	◯	◯	◯	V. Happy

26. Is the area important for spiritual or religious values? *

Mark only one oval.

1 - Yes

2 - No

27. To the best of your knowledge, does the area’s aesthetics provide you with* adequate mental wellbeing?

Mark only one oval.

1 - Yes

2 - No

28. Is the area important for jobs or job creation associated with biodiversity* maintenance and protection (e.g. working in the protected area)?

Mark only one oval.

1 - Yes

2 - No

29. Do you have any other suggestions or comments?

ESS & HW – Tables

30. What impact and link do Cultural ESS have on your human well-being? (Please mark once only for each column.)

Tick all that apply.

	Recreation	Tourism and Ecotourism	Landscape/aesthetic beauty	Education/ interpretation	Scientific research
Has had a negative impact on my well--being	☐	☐	☐	☐	☐
Does not contribute to my well--being	☐	☐	☐	☐	☐
Does nott conttrriibutte tto my wellll--beiing butt has pottenttiiall tto	☐	☐	☐	☐	☐
Makes a miinorr conttrriibuttiion tto wellll--beiing	☐	☐	☐	☐	☐
Makes a majjorr conttrriibuttiion tto wellll--beiing	☐	☐	☐	☐	☐

Q49. What impact and link do Cultural ESS have on your human well-being? (Please mark once only for each column.)

1 – Recreation
2 – Tourism/Ecotourism
3 – Landscape/Aesthetic
4 – Education/Interpretation
5 – Scientific Research
6 – Traditional Knowledge
7 – Ecological Knowledge
8 – Cultural Heritage

Q50. Which cultural services do you think have degraded? (please mark the appropriate ones)

1 – Not degraded
2 – Somewhat Degraded
3 – Degraded
4 – Severely Degraded

Q51. Which cultural services do you think have degraded? (please mark the appropriate ones)

1 – Not Threatened
2 – Somewhat Threatened
3 – Threatened
4 – Severely Threatened

Q52. To the best of your knowledge, what are the drivers of change in your area?

1 – Poverty/Economic Influence
2 – Lack of Technology
3 – Legislation/Politics
4 – Habitat Loss/Degradation
5 – Climate Change
6 – Cultural Activities

Q53. What impact and link do Provisional ecosystem Services have on your human wellbeing?

1 – Has had a negative impact on my well-being
2 – Does not contribute to my well-being
3 – Does not contribute to my well-being but has potential to
4 – Makes a minor contribution to well-being
5 – Makes a major contribution to well-being

Q54. Which provisional services do you think have degraded?

1 – Not degraded
2 – Somewhat Degraded
3 – Degraded
4 – Severely Degraded

Q55. To the best of your knowledge, what are the drivers of change in your area?

1 – Poverty/Economic Influence
2 – Lack of Technology
3 – Legislation/Politics
4 – Habitat Loss/Degradation
5 – Climate Change
6 – Cultural Activities

31. Which cultural services do you think have degraded? (please mark the appropriate

ones)

Tick all that apply.

	Severely Degraded	Degraded	Somewhat Degraded	Not degraded
Recrreattiion	☐	☐	☐	☐
Tourriism/Ecottourriism	☐	☐	☐	☐
Landscape/Aestthettiic	☐	☐	☐	☐
Educattiion/IInntterrpprrettattiion	☐	☐	☐	☐
Sciienttiific Researrch	☐	☐	☐	☐
Trradiittiionall Knowlledge	☐	☐	☐	☐
Cullttuurrall Herriittage	☐	☐	☐	☐
Ecollogiicall Knowlledge	☐	☐	☐	☐

32. To what extent do you believe cultural ESS are threatened in your area? (Please mark the appropriate ones)

Tick all that apply.

	Not Threatened	Somewhat Threatened	Threatened	Severely Threatened
Recrreattiion	☐	☐	☐	☐
Tourriism/Ecottourriism	☐	☐	☐	☐
Landscape/Aestthettiic	☐	☐	☐	☐
Educattiion/IInntterrpprrettattiion	☐	☐	☐	☐
Sciienttiific Researrch	☐	☐	☐	☐
Trradiittiionall Knowlledge	☐	☐	☐	☐
Cullttuurrall Herriittage	☐	☐	☐	☐
Ecollogiicall Knowlledge	☐	☐	☐	☐

33. To the best of your knowledge, what are the drivers of change in your area?

Tick all that apply.

	Technology	Globalization	Policy	Legislation	Natural environment
Recreation	☐	☐	☐	☐	☐
Tourism/Ecotourism	☐	☐	☐	☐	☐
Landscape/Aesthetic	☐	☐	☐	☐	☐
Education/Interpretation	☐	☐	☐	☐	☐
Scientific Research	☐	☐	☐	☐	☐
Traditional Knowledge	☐	☐	☐	☐	☐
Cultural Heritage	☐	☐	☐	☐	☐
Ecological Knowledge	☐	☐	☐	☐	☐

34. What impact and link do Provisional ecosystem Services have on your human wellbeing? (Please mark once only for each column.)

Tick all that apply.

	Has had a negative impact on my well-being	Does not contribute to my well-being	Does not contribute to my well-being but has potential to	Makes a minor contribution to well-being	Makes a major contribution to well-being
Crrop Prroducttiion	☐	☐	☐	☐	☐
Liivesttock	☐	☐	☐	☐	☐
Soiill Conserrvattiion	☐	☐	☐	☐	☐
Watterr Yiielld	☐	☐	☐	☐	☐
Sand Fiixattiion	☐	☐	☐	☐	☐
Carrbon Sequesttrrattiion	☐	☐	☐	☐	☐
Habiittatt	☐	☐	☐	☐	☐
Natturre	☐	☐	☐	☐	☐
Landscape Recrreattiion	☐	☐	☐	☐	☐

35. Which provisional services do you think have degraded?

Tick all that apply.

	Severely Degraded	Degraded	Somewhat Degraded	Not degraded
Crrop Prroducttiion	☐	☐	☐	☐
Liivesttock	☐	☐	☐	☐
Soiill Conserrvattiion	☐	☐	☐	☐
Watterr Yiielld	☐	☐	☐	☐
Sand Fiixattiion	☐	☐	☐	☐
Carrbon Sequesttrrattiion	☐	☐	☐	☐
Habiittatt Qualliitty	☐	☐	☐	☐
Landscape Recrreattiion	☐	☐	☐	☐

36. To the best of your knowledge, what are the drivers of change in your area?

Tick all that apply.

	Technology	Globalization	Policy	Legislation	Natural environment	Cultural Activities
Crrop Prroducttiion	☐	☐	☐	☐	☐	☐
Liivesttock	☐	☐	☐	☐	☐	☐
Soiill Conserrvattiion	☐	☐	☐	☐	☐	☐
Watterr Yiielld	☐	☐	☐	☐	☐	☐
Sand Fiixattiion	☐	☐	☐	☐	☐	☐
Carrbon Sequesttrrattiion	☐	☐	☐	☐	☐	☐
Habiittatt Qualliitty	☐	☐	☐	☐	☐	
Landscape Recrreattiion	☐	☐	☐	☐	☐	
